# Is it feasible to deliver a complex intervention to improve the outcome of falls in people with dementia? A protocol for the DIFRID feasibility study

**DOI:** 10.1186/s40814-018-0364-7

**Published:** 2018-11-10

**Authors:** Louise M. Allan, Alison Wheatley, Elizabeth Flynn, Amy Smith, Chris Fox, Denise Howel, Robert Barber, Tara Marie Homer, Louise Robinson, Steve Wayne Parry, Lynne Corner, Jim Anthony Connolly, Lynn Rochester, Claire Bamford

**Affiliations:** 1 Institute of Health Research, University of Exeter, South Cloisters, St Luke’s Campus, Heavitree Road, Exeter, EX1 2LU UK; 2 Institute of Health and Society, Newcastle University, Newcastle upon Tyne, UK; 3 The Newcastle upon Tyne Hospitals NHS Foundation Trust, Newcastle upon Tyne, UK; 4 Tees, Esk and Wear Valleys NHS Foundation Trust, Stockton-on-Tees, UK; 5 University of East Anglia, Norwich, UK; 6 Northumberland Tyne and Wear NHS Foundation Trust, Newcastle upon Tyne, UK; 7 Newcastle University, Newcastle upon Tyne, UK; 8 Institute of Neuroscience, Newcastle University, Newcastle upon Tyne, UK

**Keywords:** Dementia, Falls, Feasibility studies, Physical therapy, Occupational therapy, Goals

## Abstract

**Background:**

People with dementia (PWD) experience ten times as many incident falls as people without dementia. Little is known about how best to deliver services to people with dementia following a fall. We used an integrated, mixed-methods approach to develop a new intervention which combines theory generated via a realist synthesis and data on current provision and pathways, gathered through a prospective observational study as well as qualitative interviews, focus groups and ethnographic observation. This intervention is to be tested in a feasibility study in the UK National Health Service.

**Methods:**

People living with dementia in one of three geographical areas will be eligible for the study if they experience a fall requiring healthcare attention and have an informal carer. Potential participants will be identified by community services (primary care, paramedics, telecare), secondary care (ED, facilitated discharge services, rehabilitation outreach teams) and research case registers. Participants will receive a complex multidisciplinary intervention focused on their goals and interests for up to 12 weeks. The intervention will be delivered by occupational therapists, physiotherapists and rehabilitation support workers. Feasibility outcomes will include recruitment and retention, suitability and acceptability of outcome measures and acceptability, feasibility and fidelity of intervention components. PWD outcome measures will include number of falls, Montreal Cognitive Assessment (MOCA), European Quality of Life Instrument (EQ-5D-5L), Quality of Life–Alzheimer’s Disease Scale (QOL-AD), Modified Falls Efficacy Scale (MFES) and Goal Attainment Scaling (GAS). PWD outcome measures completed by an informal carer will include Disability Assessment for Dementia (DAD), EQ-5D-5L Proxy, QoL-AD Proxy and a Health Utilisation Questionnaire (HUQ). The carer outcome measure will be the Zarit Burden Interview (ZBI). An embedded process evaluation will explore barriers and facilitators to recruitment and intervention delivery.

**Discussion:**

The study results will inform whether and how a larger multicentre RCT should be undertaken. A full RCT would have the potential to show how outcomes can be improved for people with dementia who have fallen.

**Ethics and dissemination:**

The National Research Ethics Service Committee Newcastle and North Tyneside 2 approved the feasibility study.

**Trial registration:**

International Standard Randomised Controlled Trial Registry. Registration number: ISRCTN41760734. Date of registration: 16/11/2015.

**Electronic supplementary material:**

The online version of this article (10.1186/s40814-018-0364-7) contains supplementary material, which is available to authorized users.

## Background

Recent estimates suggest that there are 850,000 people living with dementia (PWD) in the UK, which will increase to over 1 million by 2025 and 2 million by 2051 if current trends remain stable [1]. While the number of people with dementia in care settings has increased, most individuals with dementia still live in the community [2]. The annual prevalence of falls in PWD ranges from 47 to 90%, depending on dementia subtype, with PWD living in their own home sustaining almost ten times more falls per year than cognitively intact older people. [3]. Their falls are more likely to be injurious than those of controls. Where injuries are sustained, PWD are less likely to recover well than other older people [4]. Falls and fall-related injuries are therefore a significant cause of morbidity and mortality in PWD.

There is presently limited evidence to guide the management of falls and fall-related injuries in people with dementia, and available evidence tends to be focused on those who sustain more serious injuries, such as fractures [5–10]. While multifactorial falls services can prevent further falls in cognitively intact older people [11, 12], their effectiveness for people with dementia has not been demonstrated [13]. Yet, there are potentially substantial benefits to be gained if the outcome of these falls and injuries in PWD could be improved. For example, a successful intervention has the potential to reduce psychological morbidity and improve wellbeing [14]. There is also evidence to suggest that rehabilitation interventions can improve physical functioning in people with cognitive impairment following hospital admission [15, 16] and that exercise may reduce the risk of future falls in this patient group [17].

The overall aim of this study is to assess whether it is possible to design a complex intervention to reduce falls in PWD living in their own homes who have sustained a fall requiring healthcare attention. The intervention was developed in earlier phases of the study (using an integrated, mixed-methods approach to develop a new intervention which combines theory generated via a realist synthesis and data on current provision and pathways, gathered through a prospective observational study as well as qualitative interviews, focus groups and ethnographic observation). We are now conducting a study to assess the feasibility and acceptability of the intervention and selected study procedures, and identify any changes needed prior to a full-scale randomised controlled trial (RCT).

## Objectives

### Primary objective

The primary aim of this study is to determine whether to progress to a full-scale randomised controlled trial which aims to reduce falls in PWD who have sustained a fall requiring healthcare attention.

### Secondary objectives

The secondary aims of this study are to determine the recruitment and retention rates from a range of settings, to identify issues related to the suitability and acceptability of outcome measures, and to assess the factors influencing the acceptability and implementation of the intervention.

## Methods

This is a single arm feasibility study of the study intervention. We have followed the Standard Protocol Items: Recommendation for Interventional Trials (SPIRIT) guidance [18] and the SPIRIT checklist is provided in Additional file 1. In developing the intervention and designing the feasibility study, we have followed the Medical Research Council (MRC) guidance on developing and evaluating complex interventions [19]. We provide a description of the intervention using the Template for Intervention Description and Replication (TiDieR) guidelines [20] in Additional file 2. In accordance with the MRC guidance on process evaluation of complex interventions [21], we have developed a logic model (to be reported elsewhere). The study will be carried out in three research sites, reflecting a range of National Health Service (NHS) practice to allow for generalisability.

### Recruitment, screening and consent

We plan to recruit a minimum of 30 people with dementia (PWD) and 30 informal carers for the intervention and up to 28 professionals for the process evaluation (beginning 5th February, ending 29th April 2018). Participants will be recruited from three geographical areas in the UK (Newcastle upon Tyne, Norwich and Stockton upon Tees).

### Inclusion criteria for people with dementia

- A known diagnosis of dementia (any subtype), made prior to entry into the study, by a specialist in dementia care (Geriatrician, Neurologist or Old Age Psychiatrist).
- Must have sustained at least one fall requiring healthcare attention (via 111 (a free-to-call single non-emergency number medical helpline operating in England and Scotland), district/practice nurse or minor injuries unit as well as the services outlined below), within 1 month prior to their identification as a potential study participant. A fall will be defined as an event whereby a person comes to lie on the ground or another lower level with or without loss of consciousness. The fall leading to their identification will be known as the index fall.
- Must be dwelling in the community at the time of the index fall and returning to the community at the time of the intervention.
- Must have an informal carer available to assist with completion of the diaries.
- Either has capacity to consent to participation, or a personal or nominated consultee who is able to give an opinion on the participation of the PWD.

### Exclusion criteria for people with dementia

- Diagnosis of dementia cannot be confirmed by the primary care team within 2 weeks of their being identified as a potential participant.
- Participant found to be dwelling in residential or nursing care, or to have been a hospital inpatient at the time of the index fall.
- Participant refuses consent, or lacks capacity and does not have personal or nominated consultee, or their personal or nominated consultee declines participation.
- Not able to communicate in English either because they are not a native English speaker or due to advanced dementia.
- Informal carer declines participation in the study.

### Identification and recruitment of people with dementia

We will compare the ease of identification of PWD via three main routes (community settings; secondary care settings; research registers); this will inform potential recruitment strategies for a future definitive trial of the developing an intervention for fall related injuries in dementia (DIFRID) intervention.

Community services used for recruitment are primary care, paramedic services and telecare services. Secondary care services used to identify potential study participants are emergency departments (ED), supported discharge teams and rehabilitation outreach teams. We will also recruit potential participants from the North East (NE) and North Cumbria (NC) Clinical Research Network (CRN) Case Register and Join Dementia Research (JDR).

At each stage of the recruitment procedure, a list of all potential participants who had contact with the research study will be maintained at each site. If the person has declined or not responded to an invitation from the research team or not been recruited for another reason then they will not receive any further contacts from the research team.

### Confirmation of PWD eligibility

With the exception of potential participants identified through primary care (who will have been identified via the Quality and Outcomes Framework (QOF) dementia register), we will have to confirm that the participant has a diagnosis of dementia prior to formal recruitment to the study. At first identification in the relevant setting, participants will be given or posted a summary participant information sheet (PIS). In community settings, participants will be asked to send in an opt in form giving their contact details. In secondary care settings, it will be possible to access contact details via patient notes. After they have received the summary, all potential participants will be contacted by the clinical trials associate (CTA) by telephone. During the initial telephone call from the CTA to discuss participation, the CTA will seek verbal consent to contact the general practitioner (GP) practice to check whether the person is on the dementia QOF register. If secondary care records indicate that the person should be on the QOF register the GP will be invited to update the QOF register to include this person.

If the participant is on the dementia QOF register, the CTA will send a full PIS. A clinical researcher will contact them again to confirm eligibility and, if still interested, to arrange a home visit to take consent and undertake a baseline assessment if appropriate. Participants who are not on the dementia QOF register will be sent a letter explaining that they are not eligible but thanking them for their interest in the study.

### Consent

Participants will be required to give informed consent to participation in the intervention study in accordance with the Declaration of Helsinki. Due to the nature of dementia, some participants may lack the capacity to give full informed consent. In this case, the provisions of the Mental Capacity Act (2005) will apply. PWD will be asked to give consent appropriate to their level of understanding, ranging from written informed consent to account being taken of verbal and non-verbal communication in determining willingness to participate. In those individuals found to be without capacity to give full informed consent, the CTA will identify a personal or nominated consultee and seek their advice regarding participation by letter and/or phone call. If a consultee thinks that the person would not have wanted to participate in the study, the participant will not be recruited and they will not be contacted any further about the study. If they do not give an opinion, it will be assumed that consent is withheld and they will not be recruited or contacted further about the study. Any PWD appearing distressed by participation or withdrawing consent will be excluded from the study without prejudice to clinical care.

### Identification and recruitment of informal carers

We anticipate that many PWD seeking healthcare attention will be accompanied by an informal carer. In this situation, the informal carer will be aware of the study from the outset. They will be issued with an informal carer PIS by the CTA at the earliest opportunity. If PWD are not accompanied by an informal carer, we will ask them to identify if they have an informal carer who might be interested in being involved in the study with them.

### Recruitment to the process evaluation

The initial consent process with PWD and informal carers will include consent for optional participation in the qualitative aspects of the study. We will purposively select a sample of consenting PWD and informal carers for observation and interview. Examples of participant characteristics which will be considered when sampling will include gender, falls history, goals and activities identified for the intervention, intensity of the intervention and adherence to the intervention (through participant diaries and discussions with the therapists delivering the intervention). We will aim to observe the delivery of all components of the intervention in all sites assuming this is logistically possible. This will enable us to explore whether and how the sessions are tailored to individuals, activities are embedded into usual routines and the role of the informal carer in the intervention.

Consent will be sought from all professionals with the exception of those involved in developing, training and supervising the intervention and those directly involved in delivering the intervention. Participation in observation and/or interviews and informal discussions will be part of their role and therefore not optional. A PIS will be provided to make these expectations clear and to outline the rationale for these qualitative aspects of the study.

To ascertain the ‘fit’ of the intervention with existing services, and the impact on referral patterns, we will liaise with the multidisciplinary team (MDT) and staff delivering the intervention to identify where referrals have been made. As part of their initial assessment, the physiotherapist and occupational therapist will explore current support services being used by the PWD. We will use information from the assessment to identify a purposive sample of staff whom we would like to interview. Selected professionals will be sent an invitation letter and PIS. This will be followed up by a telephone call within a week to check whether the professional is willing to take part and, if so, to arrange a meeting to take consent and conduct the interview.

CTAs responsible for recruitment and professionals involved in making the initial approach will be sent a PIS by the qualitative team towards the end of the recruitment period. This will be followed up by email or telephone to discuss participation and, if appropriate, arrange the interview.

### Data collection and follow up

#### Baseline assessments and data

Baseline data for the outcome measures described below will be recorded by a clinical researcher for PWD and informal carers consenting to the intervention study within 2 weeks of confirmation of eligibility (Table 1).

**Table 1:** Assessment of outcome measures

	Completed by	Time to complete	Baseline visit	12-week follow-up visit
MOCA	Patient	10 min	✓	
EQ-5D-5L	Patient	5 min	✓	✓
QOL-AD	Patient	5–10 min	✓	✓
MFES	Patient	5–15 min	✓	✓
GAS	Patient	20–40 min	✓^a^	✓^a^
TUAG	Patient	5 min	✓^a^	✓^a^
DAD	Informal carer (proxy)	15 min	✓	✓
EQ-5D-5L	Informal carer (proxy)	5 min	✓	✓
QOL-AD	Informal carer (proxy)	5–10 min	✓	✓
HUQ	Informal carer (proxy)	20 min		✓
ZBI	Informal carer	10 min	✓	✓

^a^This measure will be completed with the therapist after the initial assessment and repeated at the final intervention visit

After the baseline assessment, the clinical researcher will send a referral to the intervention team using a structured referral form with details of the baseline assessments of the PWD and informal carer. The intervention team will then arrange an initial intervention assessment within 2 weeks.

#### Follow up assessments

At 12 weeks, the clinical researcher will carry out a second visit to repeat most of the outcome measures completed at the baseline assessment with PWD and informal carers (see Table 1). The exception is the Montreal Cognitive Assessment (MOCA); completing this at baseline will enable us to describe the cognitive function of participating PWD, but it will not be repeated as the intervention is not expected to have an impact on cognition. The Health Utilisation Questionnaire (HUQ) will be completed by the clinical researcher with the informal carer on behalf of the PWD to determine the use and health and social care services by the PWD in the preceding 12 weeks.

### Outcome measures

#### PWD outcome measures

All outcome measures will be completed by those PWD with the capacity to do so. With the exception of the MOCA, all measures will be completed at baseline and 12 week follow-up (see Table 1). The schedule of events is shown in Table 2.

**Table 2:** Schedule of events

	Baseline assessment (clinical researcher)	Week 1 (intervention)	Weeks 2–12 (intervention)	Week 12 follow-up assessment (clinical researcher)
Informed consent (including consent for observation and/or interview)	X			
Baseline data collected (see Table 1)	x			
2 Assessment visits by intervention team including timed up and go test		X		
Up to 22 visits by Intervention team. Final visits will include goal attainment scaling and timed up and go test			X	
Completion of diary			X	
Informed consent of professionals and participants and observation of interventions received		X	X	
Informed consent and qualitative interview with some professionals regarding views on intervention.			X	
Qualitative interview with patients, informal carers and professionals views on intervention			X	
Follow up outcome data collected (see Table 1)				X

#### Number of falls

This will be assessed through prospective completion of a diary throughout the 12-week intervention with the aid of an informal carer when required. Participants will be asked to record whether they had any falls on each day and, if so, to describe the context and consequences of the fall. These data will be used to calculate the proportion of participants with one or more falls and the fall rate per person year.

#### Montreal Cognitive Assessment

This measure will be completed at baseline only to allow us to describe the cognitive profile of participating PWD [22].

#### Modified Falls Efficacy Scale

The psychological consequences of falling will be determined using the Modified Falls Efficacy Scale (MFES) [23]. This is a 14-item measure of falls efficacy (or fear of falling), based on the original Falls Efficacy Scale [24].

#### Goal Attainment Scaling

As part of the intervention, therapists will set individualised goals with participants. The goals will be agreed with the PWD by the therapists at the first therapy session and assigned ‘weights’. Goal Attainment Scaling (GAS) is a method of scoring the extent to which these goals are achieved in a way that is standardised for analysis [25, 26]. Progress towards goals will be measured at the final intervention visit, allowing a numerical score to be calculated at 12 weeks.

#### Generic Quality Of Life Instrument

The EuroQol-5 Dimension-5 Levels (EQ-5D-5L) is a standardised instrument used to measure generic health-related quality of life [27]. The five dimensions are mobility, self-care, usual activities, pain/discomfort and anxiety/depression. The five levels range from no problems to extreme problems.

#### Quality of Life–Alzheimer’s Disease Scale

The Quality of Life–Alzheimer’s Disease Scale (QOL-AD) is a standardised instrument for measuring quality of life for PWD [28, 29]. It is a 13-item scale administered via an interview. It includes the domains of physical condition, mood, memory, functional abilities, interpersonal relationships, ability to participate in meaningful activities, financial situation and global assessments of self as a whole and QOL as a whole.

### PWD outcome measures completed by an informal carer

With the exception of the Health Utilisation Questionnaire, all outcome measures will be completed by an informal carer at both baseline and 12-week follow up.

#### Disability Assessment For Dementia

The Disability Assessment for Dementia (DAD) is a standardised instrument measuring the functional ability of PWD in activities of daily living (ADLs) [30]. It is 40-item scale administered via an interview with a proxy.

#### EQ-5D-5L proxy

The proxy version of the EQ-5D-5L will be completed by informal carers regardless of whether or not the PWD lacks capacity.

#### QoL-AD proxy

The proxy version of the QoL-AD will be completed by informal carers regardless of whether or not the PWD lacks capacity.

#### Health Utilisation Questionnaire

This questionnaire will be completed by the clinical researchers using information from informal carers at 12 weeks to ascertain which additional health and social care services have been used by the PWD during the 12-week period of the intervention. We will use informal carers as proxy respondents since it is unlikely that many PWD will be able to provide detailed information about retrospective service use. To facilitate recall, we have included a section for health and social care appointments in the prospective diary. This will be a pilot of the questionnaire for a future definitive trial.

### Informal carer outcome measure

#### Zarit Burden Interview

Carer burden will be measured using Zarit Burden Interview (ZBI), a series of 22 questions designed to elicit the impact of the patient’s disabilities on the life of the caregiver [31]. This will be completed with informal carers at baseline and follow-up.

### DIFRID intervention

#### Description of the DIFRID intervention

**Fig. 1:**
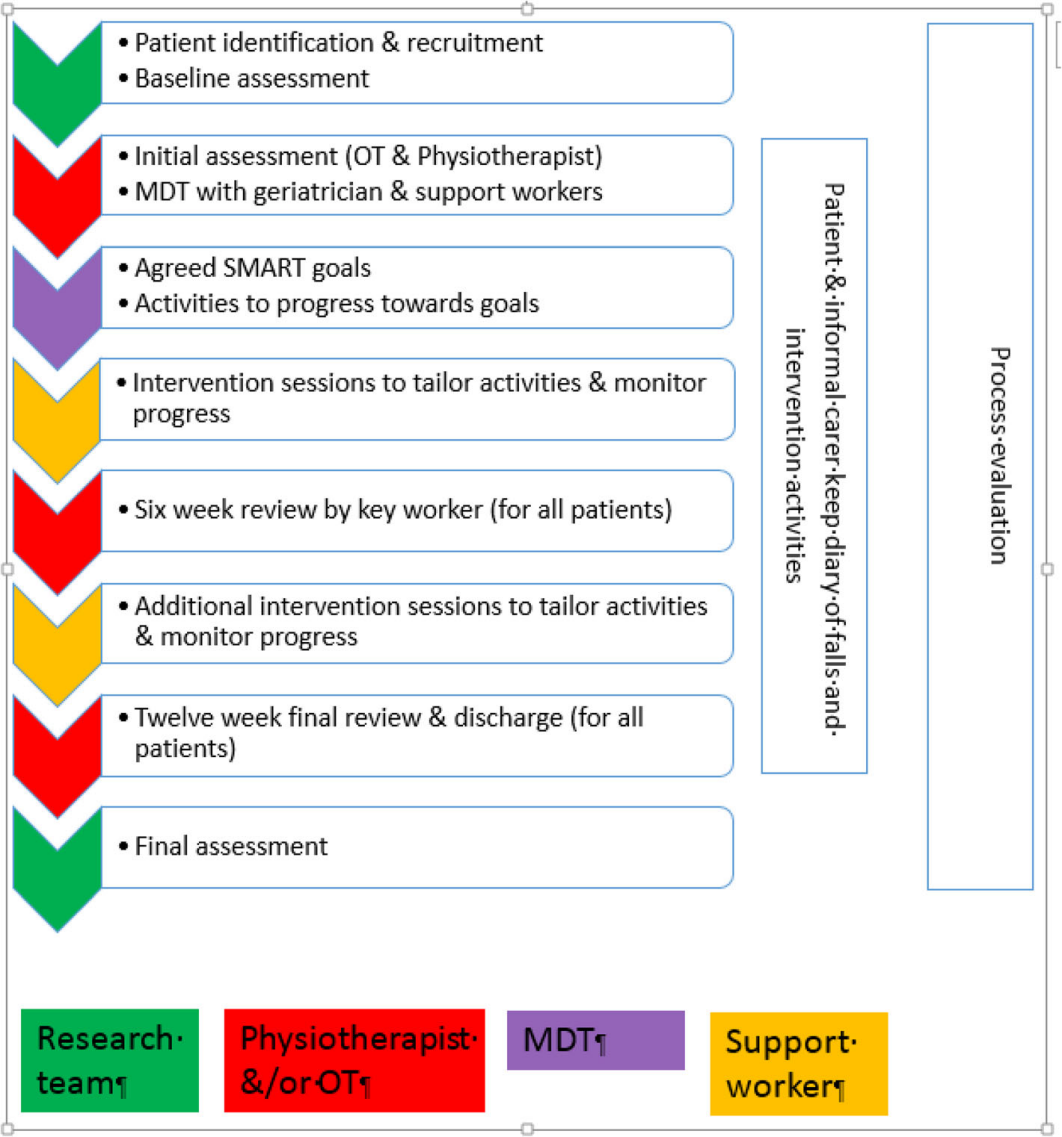
Overview of the DIFRID intervention

An overview of the intervention is provided in Fig. 1. Details of the intervention are supplied in Additional file 3. The intervention will be a multidisciplinary intervention primarily delivered in the participant’s home. The intervention will be tailored to the abilities of the participant, their likes and dislikes for activities and goals agreed between the therapist and the participant and their informal carer. The number of sessions will be tailored to the needs of the participant; the first two sessions will be assessment sessions followed by up to 22 therapy sessions delivered over a total period of up to 12 weeks. The assessment and therapy procedures are described in a bespoke manual for professionals (Additional file 4).

### Feasibility outcome measures

#### Feasibility of recruitment and retention

We will explore the feasibility of different approaches to PWD identification and recruitment. We will also consider the rates of conversion to study participation and retention. Specifically, we will report on:

- The number of PWD identified through community and secondary care, and case registers/JDR
- The proportion of PWD who give permission for us to check their medical records to determine eligibility
- The proportion of PWD who meet the eligibility criteria
- The proportion of eligible PWD who agree to participate in the study
- The proportion of eligible informal carers who agree to participate in the study
- The proportion of participating PWD and informal carers who start the intervention
- The proportion of participating PWD and informal carers who remain in the study until study completion
- The proportion of participating PWD and informal carers completing each outcome measure at baseline and 12-week follow-up

CTAs responsible for recruitment and professionals responsible for the initial approach to potential participants will be invited to take part in a formal interview to explore the feasibility and acceptability of the different approaches to patient identification.

#### Assessment of suitability and acceptability of outcome measures

We will also examine the response rates, acceptability and feasibility of outcome measures described in Table 1 that could be used in a definitive trial. We will include a small number of open questions at the final follow-up interview to explore their views on the measures used and to identify any additional outcomes of the intervention that have not been captured. The clinical researchers responsible for collecting outcome data will also be invited to take part in a brief qualitative interview to give their feedback on the outcome measures.

#### Assessment of the feasibility and acceptability of intervention delivery

Since the intervention is multi-dimensional and tailored to the individual, we will use mixed methods and seek the views of a range of stakeholders. Quantitative analysis will consider:

- The proportion of staff attending all training and supervision sessions and MDT meetings
- The number, frequency and duration of training and supervision sessions, and MDT meetings
- Time spent with the patient and time spent travelling to appointments
- The proportion of patients discussed at MDT meetings and actions taken
- The proportion of patients seen by a geriatrician
- The proportion of patients reviewed by the MDT at six and twelve weeks and actions taken
- How the assessment documentation was used in practice, for example, whether all sections were completed
- The nature of goals set and alignment of activities with these goals
- Referrals made to other services
- Adherence with agreed activities by PWD

Additional qualitative work will provide a more nuanced understanding of these data and allow us to explore whether and how the intervention will need to be adapted prior to a full trial. We will either directly observe or use audio recordings of intervention training, delivery and supervision in all sites; this will enable us to explore adherence and variation between and within sites. Observation will be supplemented with semi-structured interviews with a range of stakeholders to explore:

- The ‘fit’ of the intervention with staff usual working practices
- The acceptability of the intervention and suggested changes or improvements to the content, delivery or timing of the intervention
- Assessment of training and intervention delivery
- The feasibility and perceived value of MDT meetings
- The feasibility of different components of the intervention (e.g. goal setting and tailoring)

To ensure that we capture the views of different stakeholders, we will interview staff delivering training and supervision, staff delivering the intervention, members of the MDT, PWD and informal carers receiving the intervention and health and social care staff who are concurrently providing care to the PWD and/or to whom the PWD is referred during the intervention.

In a previous process evaluation [32], we found informal discussions to be a very effective way of collecting data in a timely fashion. Such discussions may only last a few minutes but can deepen understanding of how the intervention is being delivered. We anticipate that informal discussions will be conducted throughout the WP4 with staff responsible for training and supervision and those responsible for intervention delivery.

### Process evaluation

Interviews will explore the acceptability and perceived value of the intervention to PWD and their informal carers. Topic guides are given in Additional file 5. We will also explore the extent to which participants felt the intervention was tailored, their views on the intensity of the intervention and staff involved in delivering the intervention and any suggested changes to the intervention. PWD consenting to a qualitative interview will be interviewed separately from their informal carer where possible, but jointly if preferred by the participant. Interviews will take no longer than 60 min and will be audio recorded with participants’ permission (as documented on the initial study consent form; consent to recording will be verbally confirmed at the time of the interview). The clinical researcher undertaking baseline and follow-up assessments will include some open-ended questions to explore participants’ views on the outcome measures. These qualitative data will be recorded in detail on the case report form (CRF) and passed to the qualitative team for analysis.

As described above, professionals will be observed during intervention delivery (with the consent of the PWD and informal carer) and during MDT meetings. The qualitative team will also observe the initial training and some supervision sessions. The importance of observing supervision was highlighted in a previous study where it revealed specific areas in which additional training was required [32]. Information from the observation will inform subsequent interviews and informal discussions with professionals and will allow us to follow up emerging issues in more detail. Interviews with MDT members will explore the perceived value and sustainability of the MDT meetings as part of the intervention. Alternative models of obtaining specialist input will also be explored.

Interviews with professionals to whom PWD and/or informal carers have been referred as a result of the intervention and/or who have been providing care to participants during intervention delivery will explore their experiences of the intervention, the ‘fit’ of the intervention with the care they provide and suggested changes or improvements to the content, delivery or timing of the intervention. The appropriateness of referrals will also be explored.

### Withdrawal criteria

PWD and informal carers will have the right to withdraw from intervention delivery, outcome assessment and/or the (optional) qualitative component, without having to give a reason, although they may give one if they wish to do so. Investigator sites will clarify with participants which aspects of the study they wish to withdraw from and document this on a study withdrawal form.

The investigator may discontinue a participant from the study at any time if necessary.

Due to the nature of the disease, some participants may become very ill or die before completion of the study. Participants who withdraw from the trial will not be replaced routinely, but if an unexpectedly large number of participants withdraw early, it may be necessary to replace them to achieve adequate data to answer our research questions.

Professionals who are not directly involved in intervention supervision or delivery will also have the right to withdraw from the study at any time without having to give a reason.

### Adverse events

This is a non-drug intervention trial, using interventions that might be offered as part of a routine physiotherapy intervention. Dementia is progressive and associated with comorbidity. Inter-current illness will be very common. We will aim to achieve a balance between ensuring that any adverse events (AEs) which are likely to be related to the study are detected, and the recording of numerous unrelated events.

Falls, injuries, deaths and hospital admissions will be ascertained prospectively throughout the study. They will be recorded through diaries which will be reviewed regularly by the therapists and/or rehabilitation support workers.

We will define an AE as an incident, injury or symptom related to therapy sessions, or activities undertaken independently. The most likely AEs are fatigue, minor musculoskeletal symptoms or injuries such as muscle stiffness, or sprains, or increased falls though increased activity. Some conditions such as arthritis or angina may be exacerbated by exercise. AEs will be monitored by therapists and rehabilitation support workers, and reported where they occur. All serious AEs should be reported to the NHS Research Ethics Committee (REC).

### Analysis

#### Analysis population

All analyses will be conducted on an intention to treat basis, with sensitivity analyses used to investigate the impact of removing individuals who did not receive the intervention as allocated.

#### Quantitative analyses

The main analysis will be of feasibility outcomes. We will report the numbers of eligible participants seen over the recruitment period, and the resulting rates of recruitment, retention and data completion. Non completers will be characterised. We will also assess performance of potential outcome measures for a definitive trial. We will ascertain data completeness of the instruments and any potential bias in the completion of follow-up data to inform the choice of instruments in a future trial. The majority of the outcome data will be presented in simple descriptive tables presenting percentages, means and standard deviations.

#### Health economic data

During the intervention development work, a Healthcare Utilisation Questionnaire (HUQ) was developed and piloted—the format and administration of this questionnaire was adapted in light of the responses and feedback. The questionnaire will now be completed once by the clinical researcher at the 12-week follow-up visit with information provided by the informal carer. The clinical researcher will make notes on the CRF regarding the perceived value and burden of including space in the participant diary to make a note of services received as an aide memoire for completing the HUQ at the end of the study. These notes will be passed to the qualitative team for analysis. The data collected will be analysed and presented as completion rates and descriptive statistics; this will allow our results to be used for meta-analysis and systematic reviews.

The data will be analysed as completion rates and descriptive statistics. We will look at the overall response rates and the completion of each question in the data collection tools (HUQ and EQ-5D-5L). This will help us identify any potential issues with the data collection tools and suggest amendments for a future definitive trial. Descriptive statistics will be provided for each type of healthcare resource reported and will be presented as the mean number of visits and standard deviation.

We will also determine the feasibility of identifying and estimating costs associated with the intervention and resource use. Intervention costs will be based on the data provided by the therapists on number of sessions, travelling time, referrals and involvement of the MDT. This will determine the ease of cost collection for a full definitive trial.

#### Qualitative analyses

Field notes of observation and interview transcripts will form the formal data for analysis. Normalisation process theory (NPT) [33] will inform both data collection and analysis. This theory is increasingly being used in studies of the implementation of interventions in health care (www.normalizationprocess.org) including published studies from current applicants [32, 34, 35]. Normalisation process theory is valuable in highlighting whether problems with implementation reflect a lack of perceived relevance of the intervention (coherence); the unwillingness of participants to invest in the intervention (cognitive participation); difficulties in delivering the intervention, such as a lack of resource or shortfalls in skills and knowledge (collective action); or a lack of feedback on the impacts of the intervention or inability to adapt the intervention to meet local needs (reflexive monitoring). Identifying key barriers to implementation using NPT helps in deciding how best to optimise an intervention and associated training and documentation prior to further implementation.

All field notes and interview transcripts will be anonymised prior to analysis. Following the principles of the constant comparative method, data analysis will proceed alongside data collection. This will ensure that emerging themes and issues can be explored in subsequent data collection. The qualitative team will use data analysis workshops to consider data from different sources (observation; interviews with different stakeholders; informal discussions) and develop a coding frame. Some data analysis workshops will also include members of the extended team to offer different perspectives on the data. Once a coding frame has been agreed, we will use NVivo software to manage data analysis. The analyses will focus on issues of feasibility and acceptability of the intervention and will explore the extent to which views on the intervention are consistent within and between stakeholder groups.

#### Sample size consideration

The sample size for the intervention study was decided by the expert consensus panel. Their decision reflected their expertise as to how many participants would be needed to measure feasibility outcomes, balanced with the time for recruitment available during the available funding envelope and the likely potential recruitment rates estimated from our observational work in an earlier stage of this research programme. It is anticipated that ten participants per site will give us sufficient data to answer feasibility questions including estimation of potential recruitment rates, intervention adherence and rates of completion of data outcome tools.

### Criteria for progression to full trial

Stop/Go criteria have been developed for progression to a definitive trial.

Definite Go (‘green light’) defined as follows:

- ≥ 60% of eligible participants consenting to feasibility trial
- ≥ 80% participants attend ≥ 60% of sessions as planned
- Retention of ≥ 70% of consented participants for provision of key outcome data at 3 months
- The intervention can be delivered with fidelity, i.e. the content, frequency, duration and quality of the intervention can be delivered as set out in the intervention delivery manual
- An indication from qualitative interview and focus group work that the intervention(s) is (are) perceived as acceptable to both participants and professionals

Definite Stop (‘red light’) defined as follows:

- < 40% of eligible participants consenting to feasibility trial
- < 20% participants attend ≥ 60% of sessions as planned in a given intervention arm
- Retention of < 50% of consented participants for provision of key outcome data at 3 months
- It is clear from the process data from participants and professionals that the intervention procedures have low fidelity in terms of content, frequency, duration and quality and that they are unfeasible to deliver
- An indication from qualitative interview and focus group work that the intervention(s) is (are) not acceptable to participants and professionals

Intermediate outcomes will be defined as amber and refinement of the intervention will be undertaken in conjunction with our patient and public involvement (PPI) panel. A decision as to whether to progress to a full trial will be discussed by the Trial Oversight Committee.

### Research governance

#### Sponsor

The study will be sponsored by The Newcastle upon Tyne Hospitals NHS Foundation Trust (NUTH) (reference 8489). A formal agreement between the sponsor and each participating site, setting out the responsibilities of sponsor, chief investigator (CI) and site, including site principal investigator (PI), will be in place prior to site initiation. Evidence of local approvals including NHS organisation research and development (R&D) and Caldicott Guardian will be obtained prior to site initiation. Responsibility for study design; collection, management, analysis and interpretation of data; writing of the report; and the decision to submit the report for publication remains with the research team.

Contact information for the study sponsor:

Newcastle upon Tyne Hospitals NHS Foundation Trust

Newcastle Joint Research Office

Regent Point

Regent Farm Road

Newcastle upon Tyne

NE3 3HD

Trust.R&D@nuth.nhs.uk

#### Indemnity

The sponsor has liability for clinical negligence that harms individuals towards whom they have a duty of care. NHS indemnity covers NHS staff and medical academic staff with honorary contracts conducting the trial for potential liability in respect of negligent harm arising from the conduct of the study at site.

#### External steering committee

A Trial Oversight Committee (TOC) with 75% independent membership will provide overall supervision for a trial on behalf of the Trial Sponsor and Trial Funder and to ensure that the trial is conducted to the rigorous standards set out in the Department of Health’s Research Governance Framework for Health and Social Care and the Guidelines for Good Clinical Practice (GCP).

#### Data management

##### Data collection tools and source document identification

Data will be handled, computerised and stored in accordance with the Data Protection Act 1998. The quality and retention of study data will be the responsibility of the CI. All study data will be retained in accordance with the latest Directive on GCP (2005/28/EC) and local policy.

### Monitoring, audit and inspection

Monitoring of study conduct and data collected will be performed by a combination of central review and site monitoring visits to ensure the study is conducted in accordance with GCP. Study site monitoring will be undertaken on behalf of the study sponsor by the Newcastle Clinical Trials Unit (NCTU), in agreement with the CI. The main areas of focus will include consent and essential documents in study files. A monitoring plan will be written, agreed and signed by the sponsor and monitor.

The study may be subject to inspection and audit by NUTH under their remit as sponsor, and other regulatory bodies to ensure adherence to GCP. The investigator(s)/institutions will permit trial-related monitoring, audits, REC review and regulatory inspection(s), providing direct access to source data/documents.

The trial may be subject to audit by representatives of the sponsor or regulatory inspection. Each investigator site will permit trial-related monitoring, audits and regulatory inspection including access to all essential and source data relating to the trial.

The trial may be prematurely discontinued on the recommendation of the Trial Oversight Committee, sponsor or regulatory authority.

## Discussion

Our systematic review showed that there is currently little evidence to guide the management of falls in dementia. Nevertheless, it is an important area for research because of the high burden of adverse outcomes for people with dementia who fall and the costs to the health and social care economy. Existing falls interventions may not be suitable for PWD. Our work with stakeholders and realist review of the literature revealed that there are sufficient ideas to develop a new intervention which may address this dearth of evidence. The protocol for this study has been developed after extensive stakeholder engagement and observation of existing practices in conjunction with consultation with an expert consensus panel.

The strength of our intervention is that it takes a new approach to the problem which takes account of the differing needs of PWD and focuses on the goals which are important to them. We will be taking a holistic approach to the patient, assessing all potential needs and adapting the intervention by tailoring and embedding of activities. The activities will include both physical activities and cognitive techniques such as dual task training.

As a feasibility study, we have included robust measurement of feasibility parameters in our protocol which will enable us to determine whether it is feasible to proceed to a full randomised controlled trial. The process evaluation is a strength because this will capture detail about how the intervention is implemented and received by all relevant stakeholders. This will enable us to adapt the procedures to maximise the chances of success in any future trial.

The research question addressed in this call was identified by the Health Technology Assessment Programme (HTA) with patient and public involvement. We have shared the brief and plans for this project with older people and informal carers of PWD participating in Voice North—an organisation to facilitate the involvement of the public in research and product and service development. Voice North exists to harness the skills and experience of the public—currently over 1000 people are involved from across the North East. The participants concurred with the HTA’s view that this is an important area for research into the care of PWD. Members of Voice North also gave input into all the patient and carer facing documents.

## Conclusions

This study will provide the evidence as to whether it is possible to implement the intervention designed by the DIFRID study team in a UK NHS setting. If it is possible, then we will seek to obtain funding for a programme of research leading to a full randomised controlled trial of the intervention. Implementation of the intervention will potentially improve outcomes for PWD who fall, their carers and the health and social care economy.

## Additional files

Additional file 1: SPIRIT checklist. (DOC 121 kb)

Additional file 2: Description of the Intervention using TIDIER guidelines. (DOCX 32 kb)

Additional file 3: Description of the intervention. (DOCX 15 kb)

Additional file 4: Study manual for professionals. (DOCX 3927 kb)

Additional file 5: Topic guides for the Process Evaluation. (DOCX 29 kb)

## Abbreviations

ADLs: Activities of daily living
AE: Adverse event
CCG: Clinical commissioning group
CI: Chief investigator
CRF: Case report form
CRN: Clinical research network
CTA: Clinical trials associate
DAD: Disability Assessment for Dementia
DIFRID: Developing an intervention for fall related injuries in dementia
ED: Emergency department
EQ-5D-5L: European Quality of Life Instrument-5 Dimensions-5 levels
GAS: Goal Attainment Scaling
GCP: Good clinical practice
GP: General practitioner
HRA: Health research authority
HUQ: Health Utilisation Questionnaire
ICD: International classification of diseases
ISF: Investigator site file
JDR: Join dementia research
MDT: Multidisciplinary team
MFES: Modified Falls Efficacy Scale
NHS: National Health Service
NPT: Normalisation process theory
PI: Principal investigator
PIS: Participant information sheet
PPI: Patient and public involvement
PWD: Person or people with dementia
QOF: Quality Outcomes Framework
QOL-AD: Quality of Life in Alzheimer’s Disease
R&D: Research and development
RCT: Randomised controlled trial
REC: NHS Research Ethics Committee
TMF: Trial master file
TOC: Trial oversight committee
ZBI: Zarit Burden Interview
