# Supplementary material for: Is it feasible to deliver a complex intervention to improve the outcome of falls in people with dementia? A protocol for the DIFRID feasibility study
Source: Pilot Feasibility Stud. 2018 Nov 10;4:170. doi: 10.1186/s40814-018-0364-7 (PMC6230281; doi:10.1186/s40814-018-0364-7)
Supplement: Supplementary file 3 — Description of the intervention. (DOCX 15 kb) [file 40814_2018_364_MOESM3_ESM.docx]

**DIFRID intervention**

**Description of the DIFRID intervention**

An overview of the intervention is provided in Figure 1.Details of the intervention are supplied in additional file 3. The intervention will be a multidisciplinary intervention primarily delivered in the participant’s home. The intervention will be tailored to the abilities of the participant, their likes and dislikes for activities, and goals agreed between the therapist and the participant and their informal carer. The number of sessions will be tailored to the needs of the participant; the first two sessions will be assessment sessions followed by up to 22 therapy sessions delivered over a total period of up to 12 weeks. The assessment and therapy procedures are described in a bespoke manual for professionals (additional file 4).

**Training**

Professionals responsible for intervention delivery will receive a training session prior to enrolment of the first participant. Training will be delivered by a Consultant Geriatrician, a physiotherapist and an occupational therapist with expertise in working with PWD, supported by a qualitative researcher who will describe elements of good practice identified during the intervention development phase. Training will include information on dementia, adapting working styles to compensate for cognitive impairment and details of the intervention including assessment procedures, multidisciplinary team (MDT) meetings, goal setting and review.

**Initial Assessment sessions and MDT**

Both a physiotherapist and an occupational therapist will visit the participant during week 1. They will complete a structured holistic assessment which will include the perspectives of the participant, and their informal carer. The initial assessment will also include an exploration of their capacity and willingness of the informal carer to take part in the intervention, and their knowledge and understanding of dementia and falls (including attitudes to risk) and an assessment of carer stress (using the data from the Zarit Burden Interview as a guide). Full details of the assessment are provided in the intervention manual (additional file 4).

At the end of the assessments, a problem list will be compiled and desired goals will be discussed with the participant and their informal carer. The problem list and goals will then be finalised at an MDT meeting, and draft goals will be agreed. The MDT will also identify the need for any onward referrals including to the GP, geriatrician, mental health nurse, old age psychiatrist, continence adviser, podiatrist, optician or dietitian. The needs of informal carers, and how to address these, will also be considered during by the MDT and an action plan made where appropriate.

**Agreed SMART goals**

Goals will be agreed with the patient and carer at the first intervention session. An action plan including recommendations for activities to be carried out during therapy sessions will be formulated. One therapist will be identified as the participant’s key worker.

**Intervention sessions**

Up to 22 x 60 minute therapy sessions will be delivered over a total period of up to 12 weeks. The number and frequency of sessions will be tailored to the needs of the participant. Up to 3 sessions will be delivered by an occupational therapist and up to 3 sessions by a physiotherapist with the remaining sessions being delivered by a rehabilitation support worker.

Each session will begin by establishing rapport and checking the readiness of the PWD to engage (e.g. by assessing pain and hydration and ensuring that aids such as spectacles are available). Activities will include both physical exercises and functional activities. Physical exercises will include strength and balance exercises and dual task exercises. Activities will be embedded in their daily life e.g. practising balance exercises while standing at the sink washing up, although participants will be able to follow an exercise programme separate from their daily activities if they wish to do so. Functional activities to be included will be identified during the goal setting process and these will include encouragement to engage in community and social activities such as shopping and attending local groups. Informal carers will be encouraged to become involved in the goal setting process and in promotion of the activities, joining in with activities where appropriate. The recommendations for activity at each visit maybe supported with participant literature including pictures of physical exercises to be carried out. Participants will be encouraged to undertake increased activity throughout the day and cueing cards will be used to embed activities in daily life. A record of the activities undertaken at each visit and recommendations for activities to be performed by the participant between visits will be made using a structured proforma for each visit. The proforma will also include a review of whether the participant undertook the recommendations since the previous visit. If the participant has not adhered to the recommendations the reasons for this will be explored with the participant and goal setting will be reviewed.

Participants will not receive input from other therapy services during the trial as this may duplicate the intervention. Depending on the needs, abilities and preferences of the participant they may also be referred to other local services available for people who fall such as Staying Steady groups. After the final therapy visit the GP will be sent a summary of the interventions carried out by the intervention team and recommendations regarding ongoing service input where needed.

**Six and Twelve week review**

The goals and action plan will be reviewed and adjusted by the key worker if necessary at week 6, with a final review at week 12.
