# Supplementary material for: Is it feasible to deliver a complex intervention to improve the outcome of falls in people with dementia? A protocol for the DIFRID feasibility study
Source: Pilot Feasibility Stud. 2018 Nov 10;4:170. doi: 10.1186/s40814-018-0364-7 (PMC6230281; doi:10.1186/s40814-018-0364-7)
Supplement: Supplementary file 4 — Study manual for professionals. (DOCX 3927 kb) [file 40814_2018_364_MOESM4_ESM.docx]

#
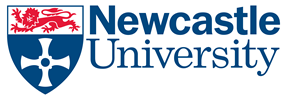


# Developing an Intervention for Falls in Dementia (DIFRID)

# Intervention Manual

# **DIFRID Intervention Manual**

Contents

[**DIFRID Intervention Manual 1**](#_Toc501464851)

[**1 Introduction 2**](#_Toc501464852)

[**2 What is the DIFRID intervention? 4**](#_Toc501464855)

[**3 Working effectively with people with dementia 6**](#_Toc501464856)

[**4 Participant identification, baseline assessments & referrals into the study 9**](#_Toc501464861)

[**5 Assessment 12**](#_Toc501464865)

[**6 Multidisciplinary team (MDT) meeting 21**](#_Toc501464869)

[**7 Agreeing goals and introducing the project diary 22**](#_Toc501464872)

[**8 Intervention sessions 27**](#_Toc501464875)

[**9 Six- and twelve-week reviews 31**](#_Toc501464881)

[**10 Consent, withdrawal and adverse events 33**](#_Toc501464884)

[**11 Final participant assessment by research team 35**](#_Toc501464888)

[**12 Process evaluation 36**](#_Toc501464889)

[**Appendix 1: Contact details of research team 38**](#_Toc501464893)

[**Appendix 2: Case report form 39**](#_Toc501464898)

[**Appendix 3: Assessment document 42**](#_Toc501464899)

[**Appendix 4: Consensus Statements 75**](#_Toc501464900)

## 1 Introduction

Welcome to the DIFRID intervention manual. This has been written for staff involved in delivering the intervention—occupational therapists, physiotherapists, and rehabilitation support workers—and members of the multidisciplinary team (MDT) supporting the intervention.

We hope the manual will be a useful reference guide alongside the training you will receive. The manual covers:

- Why a new intervention is needed for people with dementia (PWD) after a fall
- An overview of the intervention and how it was developed
- An introduction to dementia as well as practical strategies and ‘top tips’ for working with PWD
- A description of how participants will be identified for the pilot study and referred to you
- A step by step description of the intervention
- Your role in providing feedback on the intervention.

The pilot study is taking place in three geographical areas – Newcastle, Norfolk, and Stockton and North Tees. If you have any questions, you can discuss these either with members of the local team or the project team (based in Newcastle). Contact details for all members of the team are provided in Appendix 1. Delivering a new intervention can be challenging as it may require you to adapt your usual practice and learn new skills; we understand that this sometimes feels uncomfortable and you may also be anxious about whether you are delivering the intervention ‘properly’. Please feel free to share any such worries with the team; this is a pilot study to see whether it is feasible to deliver the new intervention, so letting us know of any concerns will help us to adapt the intervention (or the manual and training) to make it more workable in practice.

### 1.1 Why is a new intervention needed for people with dementia who fall?

There are three key reasons for developing an intervention specifically for PWD who fall. First, falls are very common in PWD; depending on the dementia subtype between around 47% and 90% of PWD will have at least one fall in a twelve month period. PWD living in their own homes are around 10 times more likely to fall than controls, and their falls were more likely to result in an injury. Secondly, following a fall PWD are less likely to recover well, are more likely to be hospitalised, are hospitalised for longer and are more likely to require increased care than cognitively intact older people. Finally, there is little evidence on how best to provide services to PWD following a fall. Previous post-fall interventions aimed at PWD have generally demonstrated little benefit beyond minor improvements to some quality of life indicators (which did not always reach statistical significance).

### 1.2 How was the DIFRID intervention developed?

The DIFRID intervention has been informed by two literature reviews, as well as preliminary qualitative and observational studies (Figure 1). The first literature review looked at existing evidence from clinical trials about rehabilitation for PWD following a fall; however, the number of studies was small and it was difficult to draw any firm conclusions. The second review used a “realist” approach, which meant that we could draw on a broader range of studies to develop theories about what approaches might be most appropriate for PWD and why. The qualitative studies included interviews and focus groups with clinicians, PWD and informal carers to explore their views on existing services and their ideas for service development. An observational study examined the numbers of PWD who might potentially benefit from a new intervention and the range of services they currently used following a fall.

Each of the above pieces of work was summarised and presented to an expert consensus panel who reviewed our findings and discussed:

- the most appropriate settings for recruiting participants to receive the intervention
- intervention components
- the most appropriate setting for delivery of the intervention
- professionals required and their training needs, and
- outcomes to be measured.

After the first meeting we used a modified Delphi panel approach to achieve consensus [5]. We drafted a series of statements based on the findings and subsequent discussion and sent this to members of panel in the form of a survey, asking them to indicate whether they agreed or disagreed with each statement. After two rounds of the survey, consensus had been reached on most areas. Using the results of the surveys, we conducted further qualitative interviews and focus groups to explore the acceptability of the proposed intervention with a range of stakeholders, including health and social care professionals, PWD and informal carers. The findings of these interviews and focus groups were then presented at a second meeting of the expert consensus panel where they reviewed the findings and came to a final agreement. The list of consensus statements are given in Appendix 4.


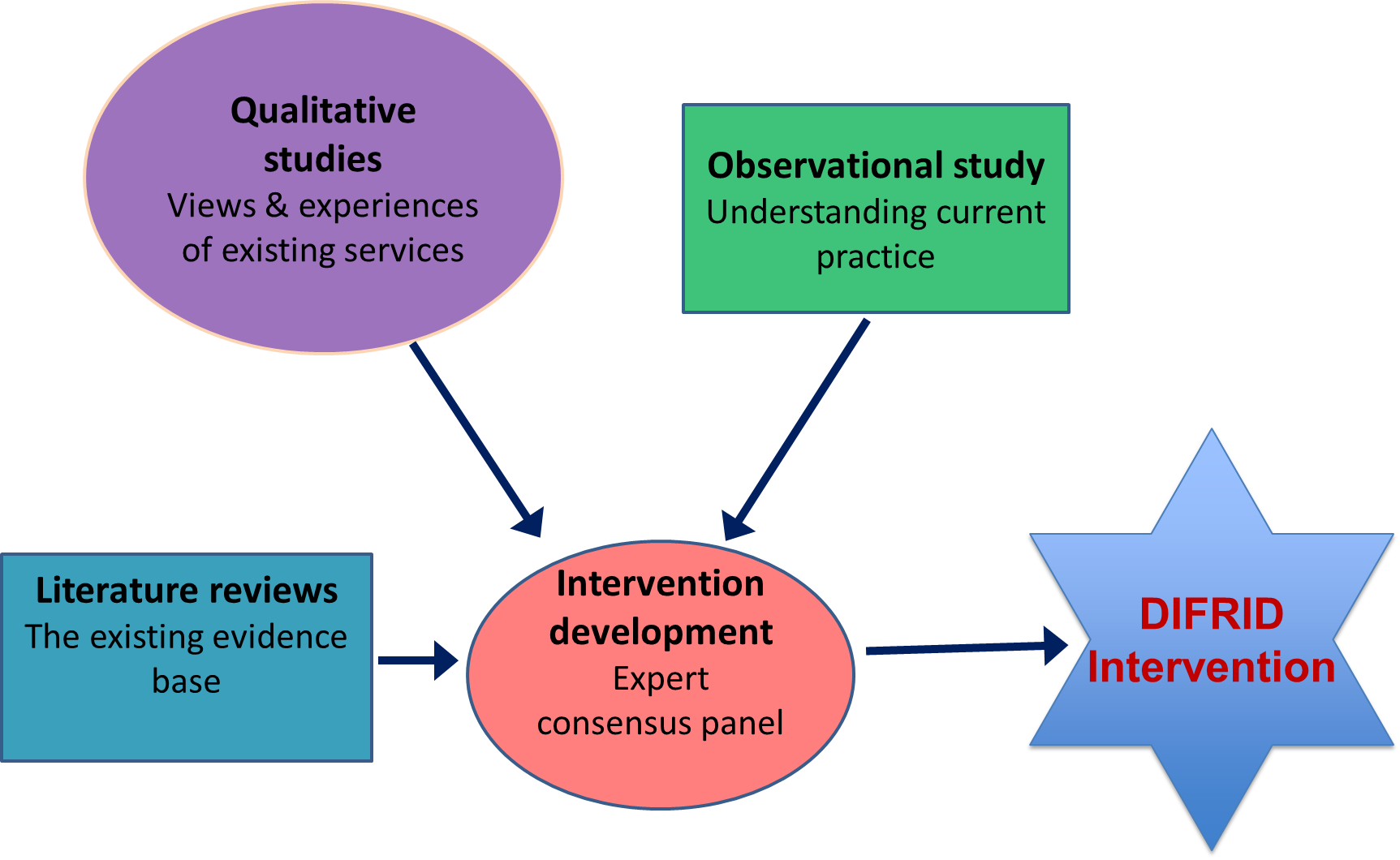
**Figure 1: Development of the DIFRID intervention**

## 2 What is the DIFRID intervention?

Figure 2 shows an overview of the intervention and includes research activities to enable you to understand the exact sequence of events experienced by each participating PWD. Section 4 onwards systematically describes each step in Figure 2, explaining both your role and that of the researchers.

**Figure 2: Overview of the DIFRID intervention**

Research team

Physiotherapist &/or OT

MDT

Support worker

Patient & informal carer keep diary of falls and intervention activities

Process evaluation

The DIFRID intervention is based on three underlying principles, which we will link back to throughout this manual:

- **Ensuring that the circumstances of rehabilitation are optimised for PWD**
- **Compensating for the reduced ability of PWD to self-manage**
- **Equipping the workforce with the necessary skills and information to work with PWD**

We developed these principles from evidence gathered during the literature reviews, qualitative studies and observational study. These principles inform all aspects of the intervention from initial assessment to final review, as described below:

**Ensuring that the circumstances of rehabilitation are optimised for PWD** means:

- **Assessing all aspects of a person with dementia’s health, not just their dementia**. Some professionals told us that dementia can mean other health problems get overlooked, such as foot care or water infections.
- **Paying attention to basic needs such as food and drink, comfort, and pain relief.** If PWD are hungry, uncomfortable or in pain, it will be harder for them to take an active role in the intervention. Delivering the new intervention in the home should help PWD feel more comfortable as they are in a familiar environment. Exploring the best time of day for visits will also help PWD to get the most out of the intervention.
- **Ensuring carers have adequate support.** Studies have consistently shown that informal carers of PWD have high levels of stress and anxiety. It is often useful for carers to help PWD with exercises or other activities. However, a key component of the intervention is to assess carer stress and signpost them to additional support if needed. This will ensure that if carers are involved in the intervention, this is not seen as another burden.

**Compensating for the reduced ability of PWD to self-manage** means:

- **Making use of multiple sources of information, including carers**. PWD may find it difficult to give an accurate account of a recent fall or may not even remember falling. Carers may be able to fill in some of the gaps and additional ideas are given to facilitate history-taking in Section 5 on assessment.
- **Designing exercise programmes to fit current routines and preferences,** by linking activities or exercises with daily routines such as opening the curtains in the morning**.** This can help exercises to more easily become habits. The frequency of sessions should also be tailored to individual needs. We aim to build on individual likes or dislikes to develop a programme of activities that is enjoyable and relevant.

**Equipping the workforce with the necessary skills and information to care for this patient group** means:

- **Involving staff with different specialities and expertise.** Each PWD will be reviewed by an MDT, which will include experts in dementia as well as physiotherapists, OTs, and rehabilitation support workers.
- **Training staff to help them give appropriate care to people with dementia.**  Having a flexible approach and communicating effectively are vital when working with PWD. Staff also need to have a good understanding of dementia, which is not always covered in detail in generic training.

## 3 Working effectively with people with dementia

### 3.1 What is dementia?

Dementia is the term used to describe a group of conditions which affect the brain and cause a decline in abilities. There are an estimated 800,000 people with dementia in the UK and this number is expected to double by 2040.

Common symptoms of dementia include changes in:

- - **Memory**
  - **Executive function.** This includes planning, organising, sequencing, and abstract thoughts. This may result in difficulties initiating or sequencing tasks.
  - **Impulse control.** This includes difficulty recognising potential risks.
  - **Problem solving**
  - **Ability to perform activities of daily living**
  - **Mood**
  - **Behaviours**
  - **Visual and spatial abilities.** This may include difficulty judging distances even where their eyesight is otherwise fine. Visual field can reduce as dementia progresses.
  - **Language skills**. This may include finding the right word to say or keeping up with a conversation. Preserved skills are linked to rhythm, which includes social chit chat.

Dementia is a degenerative condition; changes are often small to start with but will worsen over time to have an impact upon the person’s day to day life. It is not possible at present to predict the rate of decline as this varies between conditions and the person. How dementia affects an individual will depend upon the areas of the brain that are damaged and the type of dementia they have. To experience what these changes might feel like for the person with dementia, visit <http://awalkthroughdementia.org/>

### 3.2 Types of dementia

**Alzheimer’s dementia** is the most common type of dementia, affecting 62% of those diagnosed. It usually develops slowly over several years. Nerve cells die in areas of the brain responsible for storing and retrieving new information, so changes in a person’s ability to recall new information can be the first symptom. Longer term memories are initially preserved. A person with Alzheimer’s disease is likely to have a decrease in language skills (especially naming and understanding what is being said) and impairments in executive function and wayfinding. They may present as more impulsive or indecisive due to changes in their ability to problem solve.

**Vascular dementia** is the second most common type of dementia and accounts for about 20% of all cases of dementia. It is characterised by a ‘step-wise’ progression which can cause sudden changes and can fluctuate with good and bad days. Its onset is sometimes clearly linked to a cerebral event. Symptoms depend on which areas of the brain are affected. The most common symptoms are a slowing in thinking and problem solving, reduced concentration, and problems with executive function.

**Lewy body dementia (LBD)** is the third most common type of dementia and accounts for around 15-20% of all cases of dementia. It shares symptoms with both Alzheimer’s disease and Parkinson’s disease. Symptoms can include impairment of executive function, which can impact on social and daily function, visual hallucinations or delusions, and sleep problems, including vivid dreams. LBD can result in problems with movement, and therefore is associated with a higher risk of falls as well as a reduction in fine motor skills. Fluctuations in abilities can be very inconsistent.

**Frontotemporal dementia**, sometimes called Pick’s disease. This is a rarer type of dementia, affecting less than 5% of people with dementia. However, it is the second most common type for people under the age of 65. Memory may present within normal range initially with more notable changes in behaviour and personality, as well as unexpected emotional shifts. Due to the area of the brain being affected, people with this type of dementia are likely to have changes in executive function. They may also be impulsive.

### 3.3 Strategies for working with people with dementia

To give people with dementia the best chance at rehabilitation, our principles state that the environment should be optimised for people with dementia and strategies introduced to compensate for their reduced ability to self-manage. Some examples of specific strategies are given in this section.

#### Compensating for reduced language skills

When communicating with people with dementia, you should pay attention to how you speak. Changes in a person’s ability to process language may mean that tone of voice holds more meaning. Use a friendly tone and avoid jargon or complex phrases. Be prepared to have the same conversation several times. Do not draw attention to repetition.

Allow extra time between responses and give a visual as well as a verbal cue where possible. Non-verbal skills can be used to show interest and encouragement. You should make sure that you are sitting in the person’s field of vision and that the person is wearing glasses and/or hearing aids as required.

#### Compensating for changes in executive function

People with difficulties with executive function may require assistance to start tasks and prompts to continue them. You may need to break task down into chunks and give one chunk at a time. Limit distractions such as noise from the TV.

#### Making activities meaningful

When introducing a new task or activity, try to link it to something already in the person’s routine. An example could be an activity to be carried out after reading the morning paper; place activity sheets in the location that person reads the paper and include a prompt for them to complete the activity. This will serve as a memory strategy, as the person is more likely to complete a new task if it is anchored to a current one.

Tasks should also have an aim that is clear to the person and a tangible result. For example, if the overall goal is to increase activity tolerance in order to be able to prepare a meal, a person may be more agreeable to making all hot drinks within the home rather than performing unintuitive exercises.

#### Compensating for changes in memory

Keep things in familiar places rather than moving them around.

Reminders should be placed in an area that the person goes to regularly and made easily visible. Using brightly coloured paper can help.

Give written information as well as verbal if the person needs to recall specific details. You should give enough information that the person can understand what needs to be done but be as concise as possible.

#### Addressing visual/spatial difficulties

If the person bumps into furniture, you may need to suggest removing any obstacles below the knee (such as coffee tables) or placing an item on top to improve visibility. A tall vase, for example, placed on a side table will bring the table into the visual field.

Highly polished floors can look wet and changes in colour of flooring can look like a step. Big patterns on flooring, bedding, tablecloths etc. can look like there is something there that could be picked up or that needs to be stepped over or around, increasing falls risk.

You may also consider using highlighted colours or contrasts to emphasize areas. People with dementia may find it difficult to see things if they are the same colour as the background. A white toilet seat in a mainly white bathroom, for example, may make it difficult for people to distinguish where to sit down. Similarly, hand rails that are the same colour as the walls will be difficult to see and grab.

Make sure that rooms are well-lit. Older people need an increased light level due to physiological changes in the eye. Due to difficulties interpreting the visual signals sent to the brain, it is even more important that higher light levels are used for a person with dementia.

### 3.4 Further resources

Information on dementia:
<https://www.alzheimers.org.uk/info/20009/treatments?gclid=EAIaIQobChMIw8mLqPyB1wIVRzwbCh3EtQfIEAAYAiAAEgJbyfD_BwE>

Creating a dementia friendly environment:
<http://dementia.stir.ac.uk/design/good-practice-design-dementia-and-sight-loss>

## 4 Participant identification, baseline assessments & referrals into the study

This section explains the procedures that PWD will have gone through prior to their first contact with you.

### 4.1 Identification and recruitment of participants

Patients with a diagnosis of dementia and a recent fall requiring healthcare attention will be eligible for the study; this may include fractures, soft tissue injuries, or being sufficiently shaken up by the fall to merit a review by a practice nurse or GP, even if there is no obvious injury.

Potential participants will be identified by a range of services including:

- Primary care
- Paramedics
- Telecare services
- Emergency departments
- Supported discharge teams
- Rehabilitation outreach teams

Patients with a fall requiring healthcare attention and with a possible diagnosis of dementia who present to any of these services will be eligible if they are resident within the postcodes served by participating CCGs. We will also recruit potential participants from the North East and North Cumbria CRN Case Register and Join Dementia Research. This will allow PWD who have previously expressed an interest in taking part in research studies to enrol into the study if they meet the eligibility criteria.

The process of recruitment for all participants is similar, although the exact steps included will depend on how the PWD was identified. Essentially, for most PWD, the process will follow that shown in Figure 3.

**Figure 3: Overview of recruitment process**

To ensure that we include PWD with varying levels of cognitive impairment, it is not essential for participants to have the capacity to consent for themselves. We will seek the opinion of a personal consultee or attorney regarding the participation of PWD who do not have this capacity. All participants must, however, have an informal carer (a family member or friend) who is able to support their participation in the study.

### 4.2 Baseline assessment

A clinical researcher will complete a number of measures with the PWD and informal carer at the baseline assessment, most of which will be repeated at the end of the intervention (see Section 11). One aim of the pilot study is to help us determine which of these measures are most acceptable to PWD and informal carers and which are most sensitive to any changes following the intervention. A brief description of each of the measures to be completed at the baseline assessment is provided in Table 1.

| **Measure** | **Description** | **Completed by** |
| --- | --- | --- |
| **Montreal Cognitive Assessment (MOCA)** | A brief cognitive screening tool which assesses short-term memory recall; visuospatial abilities; executive function; attention, concentration and working memory; language; and orientation to time and place. | Participant |
| **European Quality of Life Instrument (EQ-5D-5L)** | A standardised instrument used to measure generic health-related quality of life for clinical and economic appraisal. | Participant |
| **Quality of Life–Alzheimer’s Disease Scale (QOL-AD)** | A standardised instrument for measuring quality of life for PWD. In addition to a global assessment of QOL as a whole, it includes the domains of physical condition, mood, memory, functional abilities, interpersonal relationships, ability to participate in meaningful activities and financial situation. | Participant |
| **Modified Falls Efficacy Scale (MFES)** | This measures the psychological consequences of falling (or fear of falling). | Participant |
| **Goal Attainment Scaling (GAS)**^1^ | This is an individualised measure which explores the extent to which goals set at the outset of therapy are achieved during the intervention. (GAS is described in detail in section 7 of the manual). | Participant |
| **Disability Assessment for Dementia (DAD)** | A standardised instrument which assesses the functional ability of PWD in activities of daily living (ADLs). | Informal carer (proxy) |
| **EQ-5D-5L (Proxy)** | The proxy version of the EQ-5D-5L which will be completed by each informal carer for the PWD they support. | Informal carer (proxy) |
| **QOL-AD (Proxy)** | The proxy version of the QOL-AD which will be completed by each informal carer for the PWD they support. | Informal carer (proxy) |
| **Zarit Burden Interview (ZBI)** | This is designed to elicit the impact of the participant’s disabilities on the life of the informal carer. | Informal carer |

**Table 1: Baseline measures**

^1^ This measure will be completed with the therapist after the initial assessment and repeated at the final intervention visit (see Sections 7 and 9).

### 4.3 Referral to the intervention team

After the baseline assessment, the clinical researcher will send the case report form (CRF) containing details of the baseline assessments of the PWD and informal carer to the team via secure NHS email. This form is included in Appendix 2.

Three of the baseline measures will include scores. These can be interpreted as follows:

#### MOCA

| 0-10 | Severe cognitive impairment |
| --- | --- |
| 10-17 | Moderate cognitive impairment |
| 18-26 | Mild cognitive impairment |
| 27-30 | Normal cognition |

#### MFES

| 0-7 | Participant has fear of falling |
| --- | --- |
| 8+ | No fear of falling |
|  |  |

#### ZBI

| 0-20 | Little or no carer burden |
| --- | --- |
| 21-41 | Mild to moderate carer burden |
| 41-60 | Moderate to severe carer burden |
| 61-80 | Severe carer burden |

An initial assessment should be arranged within 2 weeks of receiving the referral from the clinical researcher. If there are any queries about the referral, these should initially be discussed with the local clinical researcher who completed the baseline assessment.

To avoid duplication, we are keen for patients not to receive standard care and the DIFRID intervention concurrently. As soon as you receive a referral, please alert any other services which could potentially be involved that the patient's care will be provided by the intervention team and that all patients will be reviewed at the end of the intervention and referred into other appropriate services at that time.

## 5 Assessment

### 5.1 Overview

It is expected that the assessment will be completed in 2 visits, one from the physiotherapist and one by the occupational therapist. The assessment document has a generic part which should be completed at the first assessment visit, a needs list to be completed by both therapists, and a final action planning section which should be completed after the second assessment visit. There are also more specific sections for each discipline, as shown in table 2 below. A copy of the assessment document is provided in Appendix 3.

**Table 2: Sections of the assessment document**

| **Generic assessment** | |
| --- | --- |
| Consent | |
| Falls history and falls risk assessment | |
| Past medical history and comorbidities | |
| Medication | |
| Current activity levels | |
| Challenging behaviour and sleep disturbance | |
| Assessment of the needs of the informal carer | |
| Current mobility | |
| **Physiotherapy assessment** | **Occupational therapy assessment** |
| Posture and general observations of pain, sensation and tone | Details of home environment |
| Lying and standing blood pressure (BP) | Self-care and productivity |
| Range of movement | Cognition |
| Muscle power | Task observations |
| Timed Up and Go test (TUAG) | Functional difficulties relating to spatial awareness, vision and hearing |
| Needs list (page 18 of assessment document) | Needs list (page 18 of assessment document) |

The physiotherapist/OT split will depend on the usual operational activity and experience within the team. This is just a guide, as some therapy teams work more generically than others and may already have overlapping areas between OT and physiotherapy.

### 5.2 Equipment required

To complete the assessment, you will require:

- tape measure for marking out the 3 metres for the TUAG
- stop watch for timing the TUAG
- cone/marker to identify turning point at 3m
- blood pressure monitor for recording lying and standing BP

### 5.3 The assessment process

Prior to each assessment session, attention must be paid to the comfort of the participant, including nutritional needs and assessment of pain (using tools to assess non-verbal signs of pain if appropriate). There is a pain checklist on page 7 of the assessment document

#### 5.3.1 Generic assessment

The generic assessment should be completed by the therapist (either physiotherapist or occupational therapist) who makes the initial visit. More detail about each section is given below.

- **Consent**

Record how the participant consented to the trial. This information can be found on the CRF. More detail about ongoing consent can be found in Section 10.

- **Falls history with summary of the fall**

In this section the assessment, you should record:

- - Background to the circumstances of the fall and location. Include details such as time of day, where the fall occurred, whether the participant was unwell or tired, and if the fall was in a familiar location or in new surroundings.
  - Mechanism of the fall **-** trip, slip, faint, collapse
  - Whether the participant was able to get up and, if not, how they got help
  - Whether or not they have a care alarm
  - Details of services received to date and any ongoing care e.g. ambulance services, hospital admission, discharge with community support team, home care package changed, day unit support, reablement, community support team without hospital admission

Bear in mind the communication needs of people with dementia when taking falls history and consider carers as additional information sources. If possible, observe the participant navigating the space in which they fell; this can be useful in determining what happened.

- **Assessment of risk factors for falls**

This part of the assessment includes a checklist with questions to ask regarding fear of falling, nutrition, fluid intake, pain, urinary incontinence, bowel incontinence, supportive footwear, visual impairment not corrected with glasses. Information gathered in this and the previous section can help you to determine at the MDT meeting if there is a need to refer the participant to other services for support.

- **Past medical history and comorbidities**

You should use your usual procedures for checking medical history, for example GP or community records. You should also take a verbal history from the participant and the informal carer. Tick any relevant conditions on the list and add further history in space provided.

- **Details of treatment offered so far and services already involved**

If you identify any potentially conflicting interventions, please alert those services as directed on page 11.

- **Medication, including number of medications**

Record from the participant’s most recent pharmacy list, as current medication may have changed since referral to the study.

- **Current levels of activity**

Record routines and likes and dislikes for activities. This information could include preferences for social activities, favourite sports or exercise, family activities, craft clubs, lunch groups, religious groups, hobbies, musical tastes, or anything that might help with planning a programme of meaningful activities. Preferred times of day for different types of activities may also be useful in creating a person-centred programme.

- **Challenging behaviour or sleep disturbance**

There may be times of each day when the participant is less likely to engage in the activity plan, and it is helpful to be aware of this in advance. Identifying reasons for fatigue or poor concentration, including whether there is a pattern at certain times of day, should be taken into account when planning activities and the timing of intervention sessions.

- **Assessment of the needs of the informal carer**
  - Record the relationship of the carer to the participant, including whether they live together and how often they provide support.
  - Record the number of people who support the participant in a caring role.
  - Record evidence of carer burden or stress**.** Ask them how they feel and if they have any difficulties as carer. Find out how they are coping.
  - Signs of informal carer stress may include: denial, exhaustion, anger, sleeplessness, health problems, irritability, social withdrawal, lack of concentration, depression, and anxiety.
  - Record how the carer feels about assisting with the intervention. This information should be taken forward into intervention planning. It should not be assumed that the carer is willing or able to help supervise activity plans or therapy programmes.
- The Alzheimer’s Society produces a leaflet with tips for carers on looking after themselves, which you may find useful: <https://www.alzheimers.org.uk/download/downloads/id/3420/carers_-_looking_after_yourself.pdf>
- **Current mobility**

This assessment uses a system of points from 0-4, where 0 indicates the participant is able to do the task alone without difficulty and 4 indicates that the participant is unable to do the task, even with the support of two carers. For each item, note in the comments box how many carers are needed to assist as well as any adaptations used e.g. 2 rails on stairs or use of a bed rail.

1. **Bed mobility**. This includes being able to turn in bed plus bringing self to sit on edge of the bed. **Lying and standing BP can be measured during this stage of the assessment**; see details on page 15.
2. **Chair mobility.** Can the participant transfer to and from the chair from the bed?
3. **Toilet mobility.** Can the participant transfer to the toilet?
4. **Stairs**. Can the participant walk up and down stairs independently?
5. **Indoor mobility.** Note any walking aids used.
6. **Dual task mobility.** Ask the participant to walk while counting backwards from 100 in 1s then 7s. Record whether they can manage to walk without obvious reduced speed, whether their gait pattern changes, whether they need to stop, or whether they are unable to do this.
7. **Outdoor mobility.** Note any walking aids used. Also note if they report having more difficulty or needing more assistance on certain types of ground e.g. slopes, uneven ground, grass, steps.

#### 5.3.2 Physiotherapy assessment

- **General observations and posture**

Use the body chart to record general observations including pain, tone and sensation:

- **Pain**: indicate with P1 the area of most obvious pain. If there are several areas, describe using P2, P3 etc. Indicate if pain is at rest or only on movement.

As pain can be difficult for some people with dementia to communicate, an added checklist is included which gives prompts for possible signs of pain. Please put a tick against any signs of pain you notice at rest or on movement. Put a cross against those where no sign is observed.

- **Tone**: mark clearly on the body chart if there are any changes in muscle tone. This will typically be for participants with other neurological conditions such as stroke. Use a plus sign (+) for increased tone and ++ for high increased tone (where joint range is reduced due to spasticity). Use minus (-) for reduced tone with -- for flaccidity.
- **Sensation**: mark sensory impaired areas on the chart, describing if this is to touch or if there is paraesthesia or other sensory loss.
- **Lying and standing blood pressure (BP)**

Use the blood pressure monitor to record lying and standing BP. The procedure is shown in table 3. This is best done when you are assessing bed transfers if you are involved in the initial generic assessment or it can be completed during the physiotherapy assessment depending on the preferences within your local team.

**Table 3: Lying and standing BP**

| **Procedure** |
| --- |
| Ask the patient to lie down for at least five minutes |
| Measure and record BP while the patient is lying down |
| Ask the patient to stand up (with assistance if needed) |
| Measure and record BP immediately after standing |

Results should be taken to the MDT meeting for discussion with the geriatrician. It is expected that no immediate action will be needed as a result of taking this reading for assessment purposes. However, if the patient is acutely unwell with other signs and symptoms, you should follow your usual protocol for managing an acutely unwell patient.

- **Range of movement**

Record full range with a tick or use N/T if not tested. Add in ¼ ½ ¾ to show reduced ranges. Base this on your knowledge of normal ranges of movement.

- **Muscle power of main muscle groups**

Using Oxford scale:

0 = no movement available

1 = palpable contraction (no visible movement)

2 = movement but only with gravity eliminated

3 = movement against gravity

4 = movement against resistance but weaker than other side

5 = normal power

Circle left or right to show which hand is dominant. The upper limb can be tested in sitting, with lower limb best tested lying. If this is not practical, then record this.

- **Timed Up and Go test (TUAG)**

This is a 6 metre walk test incorporating a 180 degree turn. The score sheet on page 11 of the assessment document divides the TUAG into sections with comment space to record any relevant information relating to each stage. For example, you should note any stages where pain was apparent or where balance was lost and the participant needed physical support to prevent a fall.

You should also record whether a walking aid was used, the footwear the participant was wearing, and where the test was completed so that conditions can be replicated at the final assessment.

#### 5.3.3 Occupational Therapy assessment

This section includes a full environmental and functional assessment. Each section on the assessment form has subheadings with prompts to help you to provide the information needed to plan the goals and interventions with the participant. These prompts are there for guidance and you do not necessarily need to comment on all aspects.

The sections are:

- **Home environment**
- **Lighting**
  - Is it of sufficient brightness?
  - Is there availability of task-specific lighting?
  - Light levels may need to be increased to meet a participant’s needs i.e. brighter bulbs
- **Can the participant easily turn on lights and are they orientated to the location of the switch?**
  - It may be beneficial for switches to be highlighted in different colour to the wall
  - Bedside lamps, motion sensor lights, and night lights can aid orientation
- **Cleanliness**
  - Can the participant clean their home to their previous standard, including heavy tasks such as vacuuming?
- **Clutter and trip hazards**
  - Is clutter present on worktops and walkways?
  - Are there trip hazards? Consider rugs, threshstrips etc.
  - Due to reduced visual field items such as coffee tables that are below the knee are likely trip hazards—they should be removed from walkways or items should be placed on top to raise height.
- **Access to frequently used items**
  - Does the participant have to complete long reaches to retrieve items?
- **Indoor / outdoor steps**
  - Are grab rails fitted?
  - Are stairs steep or narrow?
  - Does the participant have any other conditions that may impact safety on stairs, such as one-sided weakness or foot drop?
- **Are edges of steps easily identifiable?**
  - Are steps well lit?
  - Heavily patterned carpets or shadows may make seeing edges difficult. Participants may require the edges of stairs to be in different colour.
- **Bathroom and toilet**
  - Are aids fitted already?
  - Is there a need for new adaptations?
- **Flooring**
  - Can the participant navigate changing colour in flooring?
  - Is flooring non-slip and not reflective? PWD may perceive changes in colour as steps and reflective flooring as a wet surface.
- **Location of falls—where have they occurred in the house?**
  - Are there any specific hazards at location of falls?
  - Do previous falls impact upon ability to complete tasks i.e. has the participant stopped using the bath after a fall?
- **Self care / productivity**
- **Dressing**
  - Is the participant dressing appropriately?
  - Do they sit to dress? Can they tie shoe laces and put on socks? Do they lose balance reaching forward?
  - Is there sufficient space in the bedroom?
  - Does clothing present trip hazards i.e. dressing gowns trailing floor that need to be taken up?
- **Washing**
  - Does the participant require support or prompts?
  - Are items easily reached?
  - How do they wash? (bath, shower, strip wash etc.)
- **Urgency to use toilet**
  - Is the toilet easily accessible from bedroom, living room, and other frequently used rooms?
- **Household management**
  - Have there been any changes in the participant’s ability to complete tasks such as laundry, washing up, and cooking?
  - Can the participant stand for the duration of each task?
- **Can the participant easily carry drinks and meals between rooms if required?**
  - Is equipment required to enable this?
  - Do rugs need to be removed?
  - Is there non-slip material on trolley/tray to reduce items slipping?
- **Does the participant have pets?**
  - Can the participant bend over to feed and tend to their pet?
  - Does the participant trip over their pet? If so, consider ways to mitigate risks such as putting a bell on the pet’s collar.
- **Medication**
  - Is medication in a weekly medication pack?
  - Does the participant have the fine motor skills to access medication pack?
  - Is a medication routine being adhered to?
  - Is the participant orientated to the day? Does this match pack / amount of medication left in pack?
  - Are they supported with medication?
- **Affect**
- **Is the participant motivated to take part in the assessment/intervention?**
  - Note their level of interest in occupations, sense of purpose, and level of satisfaction.
- **Does the participant lack confidence?**
  - Note expectation of success and self-esteem.
- **Coping mechanisms**
  - Has the participant put compensatory strategies in place?
  - Are these meeting their needs?
- **Conversation rate and tone**
  - Note vocal expression, body language/non-verbal cues, conversation, assertion, anxiety.
- **Does the participant have a sense of purpose and role?**
  - Have they experienced a loss of role?
  - Relationships
- **Cognition**
- **MOCA score (see referral document)**
- **Orientation to home environment and local area**
  - Note orientation (time, place, person), memory, concentration, attention, abstract/concrete thought, ability to make choices, level of arousal/alertness, ability to maintain on topic of conversation.
- **Orientation to appointments**
  - Does the participant miss appointments? Consider memory strategies to recall appointments.
- **Skilled observation of task**
  - Note functional movements e.g. reaching, carrying and bending.
  - Observe ability to concentrate, follow instruction, and identify and rectify errors.
  - Note planning, sequencing, and ability to identify and use items for task appropriately.
  - Can the participant follow verbal instructions and recall information after short delay?
  - Are they orientated to the location of items?
- **Awareness of falls risk and impact on activities of daily living**
- **Insight into falls risk and loss of abilities**
  - Can the participant identify risk?
  - Is this realistic?
- **Has the participant withdrawn from activities due to fear or perceived risk?**
  - Have they stopped going out?
  - Is there increased hesitation when out? e.g. holding tightly onto others
  - Will participant only complete tasks when someone else is present?
  - Does their fear impact on ADL ability and/or stop them from engaging in activity?
- **Perception / sensory impairments**
- **Spatial awareness / visual perception observation**
  - Observe participant navigating doorways, negotiating stairs (look for hesitation before placing feet), and making transfers (missing surfaces when sitting).
  - Can the participant see overlapping figures (i.e. when picking up items from drawers)?
  - Do they over or under reach when opening cupboards or picking things up?
- **Use of spectacles**
  - Note if they use multifocal lenses or glasses for reading only.
  - Does the participant have any eye conditions, such as cataracts or macular degeneration?
- **Does the participant use hearing aids?**

#### 5.3.4 Needs list

Both therapists should add to the needs list (page 18 of the assessment document) with actions for discussion at the MDT. The actions should focus on the individual’s needs, particularly any areas they identify themselves as specific areas of need.

#### 5.3.5 Action planning using compass of life

At the end of assessment visit 2, the action planning section on page 19 of the assessment document should be completed by the attending therapist. Figure 4 shows a tool called the Compass of Life, which is used to help participants identify goals which are important to them. Ask the participant to rate the importance of each category to them from 0 (not at all important) to 10 (very important). This information can then be used to help plan an individualised programme of meaningful activities according to the principles of the intervention.

Figure 4: Compass of Life

For more information about the Compass of Life, see Kampe et al (2017) ‘Hip and pelvic fracture patients with fear of falling’ <https://doi.org/10.1177/0269215517691584>


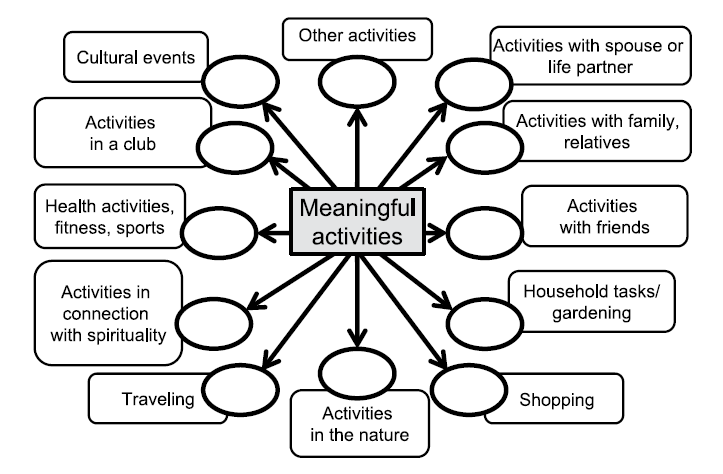


## 6 Multidisciplinary team (MDT) meeting

Following the two assessment visits, an MDT should be arranged for discussion of intervention planning for the 12 week period of the study and to decide whether any onward referrals are required, and, if so, who will make them.

### 6.1 Professionals involved in the MDT

- Physiotherapist
- Occupational therapist
- Rehabilitation support workers
- Geriatrician

### 6.2 Purpose and outcomes of the MDT meeting

The aim of the MDT meeting is to discuss the findings from the initial assessment and develop an intervention action plan based on participant and informal carer goals. Outcomes will include:

- Agreeing a proposed set of participant-centred goals based on:
- The participant’s stated goals and action plan (assessment document page 19), including the Compass of Life
- The participant’s likes and dislikes
- Informal carer needs
- The needs list produced from the assessment
- Medical interventions required

The goals suggested by the MDT should have an expected outcome defined (i.e. what success will look like for that participant). These goals will be confirmed with the participant at intervention session one and written on the Goal Attainment Scaling (GAS) form (page 23). More information about GAS can be found in Section 7.

- Agreeing the type of activities and interventions that are most appropriate for this participant in order to meet their goals. More information on this is given in Section 8.
- Agreeing onward referrals and identifying a named individual responsible for each referral e.g. local activity groups, Age UK activities, Alzheimer’s support groups, council leisure centres, day units, orthotics, wheelchair services, continence advice, specialist clinics and services. Each professional will then record when they made the referral with their name on the onward referral form (assessment document page 21).
- Agreeing the key worker for the participant. The key worker will supervise the rehabilitation support worker and will also lead on monitoring and adapting the goals with the participant and informal carer during the 12 weeks. They will be the named contact for the participant during the study.
- Agreeing the number of interventions sessions needed for the first 6 weeks and who will visit each time. This is likely to be a mix of OT (up to a maximum of 3 sessions over 12 weeks), physiotherapist (up to a maximum of 3 sessions over 12 weeks) and rehabilitation support worker. A maximum of 22 60-minute therapy sessions are available over the 12 week intervention period, but the number and frequency of sessions should be tailored to the needs of the participant. For example, sessions could be delivered evenly over the period of 12 weeks or in a tapered format such as 2-3 sessions per week for 8 weeks then weekly thereafter. The participant may need fewer than 22 sessions.

## 7 Agreeing goals and introducing the project diary

Each intervention session should follow the instructions given in Section 8. However, at the first intervention session following the MDT meeting, some additional things need to be done. This includes agreeing goals with the participant, completing the GAS form, and introducing the project diary. These are explained in more detail in the following sections.

### 7.1 Goal Attainment Scaling (GAS)

GAS is a method of measuring the extent to which a participant’s individual goals are achieved in the course of the intervention. In effect, each participant has their own outcome measure but this is scored in a standardised way to allow statistical analysis.

In GAS, tasks are individually identified to suit the participant, and the expected outcomes are individually set around their current and expected levels of performance. It is a valid individualised treatment outcome and can be used to track multiple goals. The goals can be prioritised and differentially weighted to reflect treatment objectives. Actively involving the participant in goal setting often results in better outcomes as they are motivated to participate in interventions.

A step by step guide to completing the GAS table is given below.

#### 7.1.1 Confirm goals

Discuss the suggested goals with the participant and informal carer and modify them if required. Agree what success will look like if that goal is achieved. Finalise goals, ensuring that they following the SMART principle (Specific, Measurable, Attainable, Realistic and Timely). Goals will need to be complete within the 12 week treatment timescale; they can be met before this time, however, and you would measure if they are maintained at 12 week review.

#### 7.1.2 Prioritise goals

Identify the importance of each goal and the level of difficult the participant perceives they have having in completing it currently.

| **Importance** | **Difficulty** |
| --- | --- |
| 0 = not at all (important) | 0 = not at all (difficult) |
| 1 = a little (important) | 1 = a little (difficult) |
| 2 = moderately (important) | 2 = moderately (difficult) |
| 3 = very (important) | 3 = very (difficult) |

This will guide you on which goals the participant would like to concentrate upon and how difficult they will be to achieve. If the participant is having significant difficulty in completing a task you may modify what achievement of this goal will look like for that person so that realistic expectations are set.

All goals should be scored above 1, as any lower indicates this it is not of sufficient importance to the person and will have little impact upon their wellbeing. Participants are unlikely to be motivated to work towards a goal that is not at all important to them.

#### 7.1.3 Agree expected outcome

An important feature of GAS is the prior determination of what a successful outcome for that individual would be. This is agreed with the participant and informal carer before the intervention starts. This is to ensure that goals are tailored to the participant, that everyone agrees each goal would be worth striving for, and that everyone has a realistic expectation of what is likely to be achieved. Each goal is rated on a 5-point scale, with the degree of attainment captured for each goal:

- If the participant ***achieves*** the expected level, this is scored at ***0***.
- If they achieve a ***better*** than expected outcome this is scored at:

***+1 (somewhat better)***

***+2 (much better)***

- If they achieve a ***worse*** than expected outcome this is scored at:

***-1 (somewhat worse) or***

***-2 (much worse)***

What each level will look like should be agreed within the MDT and with the participant and informal carer.

#### 7.1.4 Score baseline

This is usually rated –1, unless the participant is as bad as they could be in that particular goal area, in which case the baseline rate is –2. This is rated so change can be monitored.

#### 7.1.5 Goal Attainment scoring

Rate the outcome scores at the appointed review date. Score to which extent they have most closely met the previously agreed outcomes.

Table 4 shows a completed example GAS form.

**Table 4: Example GAS form**


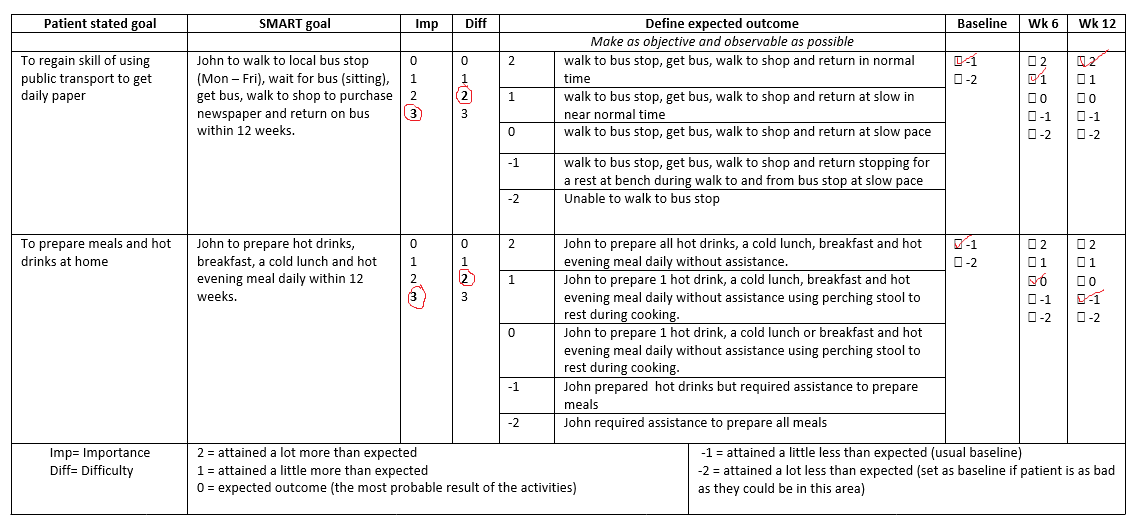


### 7.2 The project diary

PWD taking part in the intervention be asked to fill in an activity diary for 12 weeks, with the assistance of their informal carer. The diary will be used to record falls; compliance with activity recommendations between therapy sessions; and service use. This data will help us to assess the outcomes of the intervention (e.g. number of falls participants have) as well as to look at how the intervention fits within current care pathways.

The diary should be given out by the therapist at the first intervention session. It consists of several sections, some of which we will need you to help complete. The project team will provide printed diaries for you to distribute.

#### 7.2.1 Goals

Once participant goals have been agreed upon, they should be **written in the diary** in addition to the assessment document **by the person delivering the first intervention session**. This is so that participants have a record of them. The goals should be written in language the participant can understand (i.e. not necessarily in the GAS format).

#### 7.2.2 Weekly activities

We will ask participants to tick a box each day to show whether or not they have completed each activity. The list of activities should be **filled in by the person delivering the intervention** each week (see Section 8 for more information on activity planning).


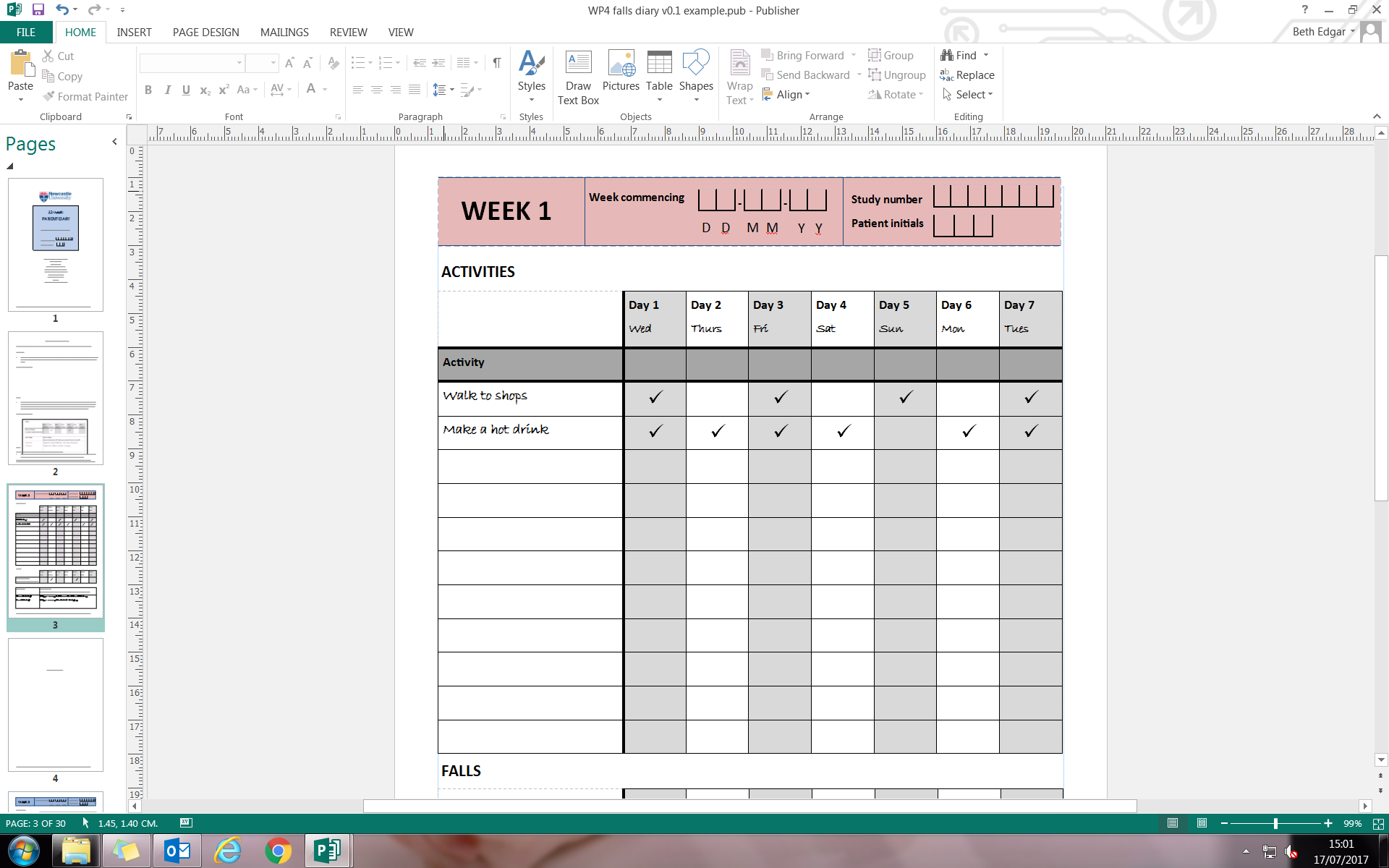


#### 7.2.3 Falls


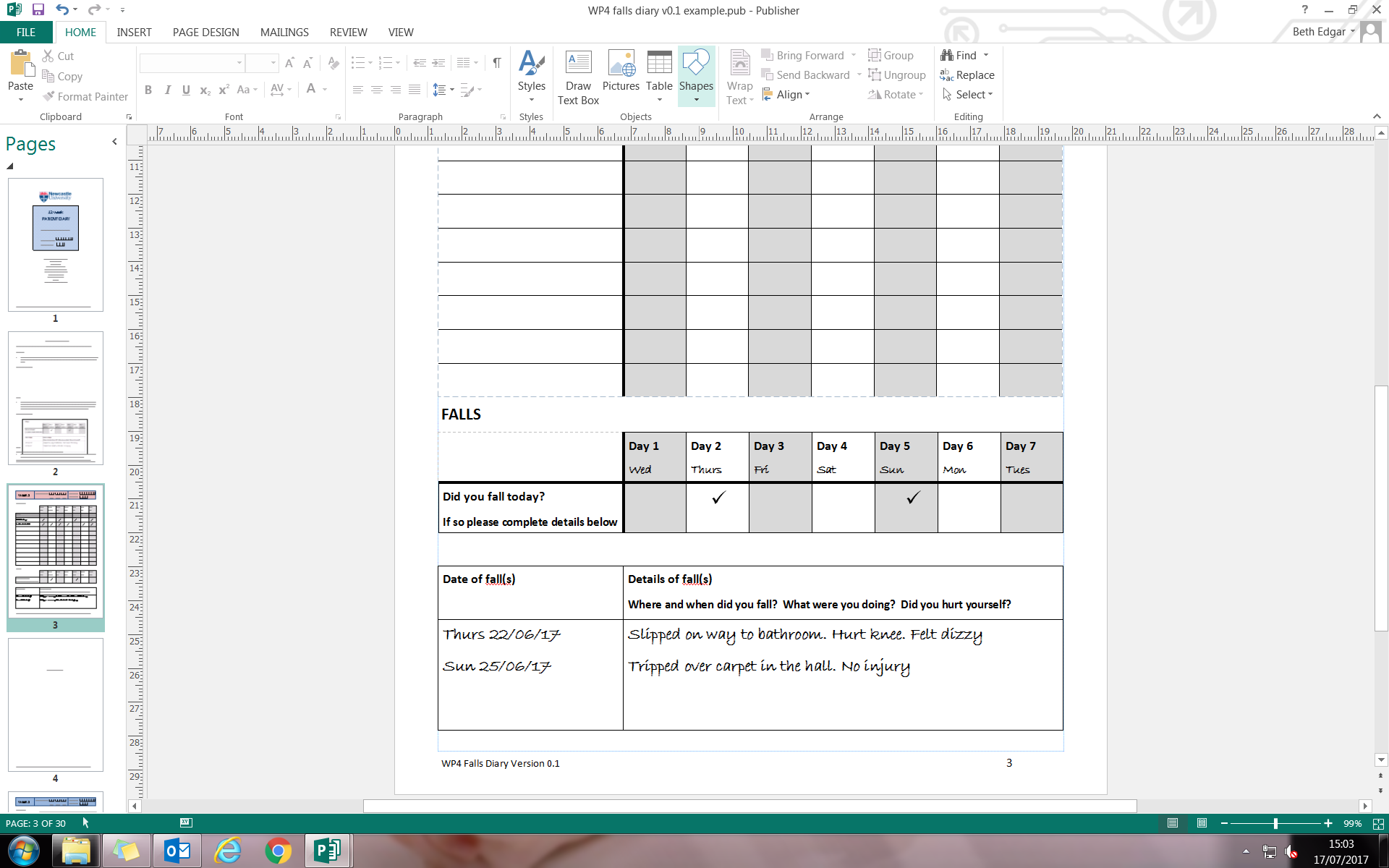
Any fall in which the participant lost his/her balance and landed on the floor or ground or lower level should be recorded in the table each week. This section should be filled in by the participant or informal carer, but you should check at each visit to help make sure the information collected is as accurate as possible. This will also enable us to record any adverse events (see Section 10).

#### 7.2.4 Service use


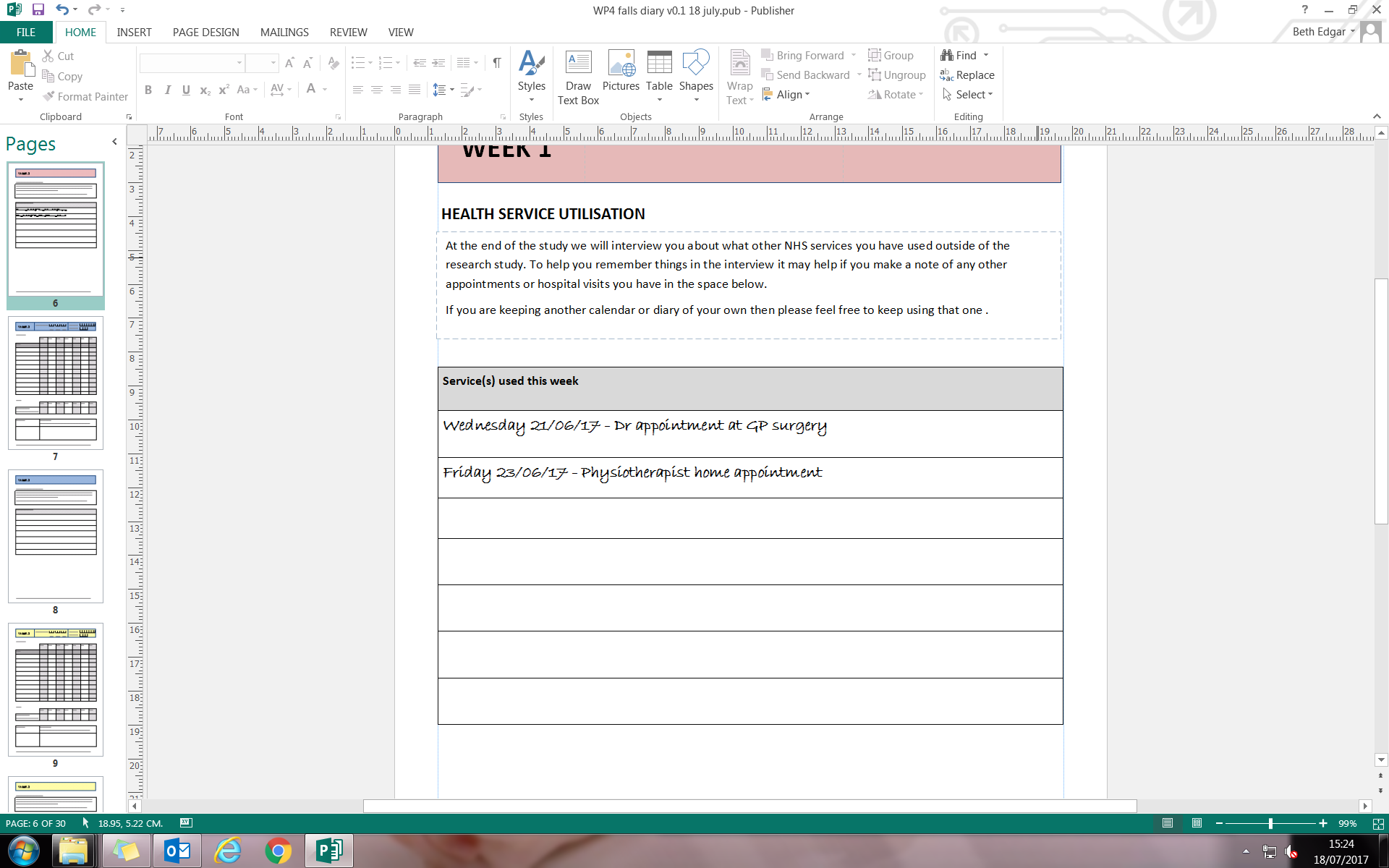
Finally, we will ask participants to record any NHS services they use which are different to their usual support package.

This part of the diary is not mandatory, and if participants are keeping another calendar or diary of your own they are welcome to keep using that one. The data will be used by the clinical researcher to help complete the Health Utilisation Questionnaire in the final interview. Again, by prompting at each visit, you can improve the quality of the information collected.

## 8 Intervention sessions

Following the two initial assessment visits and the MDT meeting, the intervention sessions will begin. The number of sessions each participant receives will be determined by their individual needs and goals, and will be agreed with the therapists and the participant. It is expected that an average of 18 intervention visits will be required per participant in addition to the two assessment visits. A maximum of 22 total visits is available over the 12 weeks.

In the first 6 weeks, more input from the team may be required, decreasing as the participant becomes more active and able to continue with home activities. Conversely, some participants may need more as the time progresses as they start to engage and adhere to the plans. Some participants will require weekly support from the therapists and rehabilitation support workers, whereas others may need twice weekly or more frequent visits, particularly if they require close supervision and prompting. Other participants may only need telephone contact or quick home checks to ensure they are attending regular exercise groups or activities to support them with the plan.

While the overall number of intervention sessions is flexible, all participants will be reviewed at 6 and 12 weeks by their key worker. This is described in Section 9.

### 8.1 Helping PWD get the most out of each session

Before, during and after each intervention session, attention must be paid to the comfort of the participant, including nutritional needs and assessment of pain (including non-verbal signs of pain). There is a pain checklist on page 7 of the assessment document if required. The participant should be comfortable before commencing any activities; remember that people with dementia may find it difficult to communicate their needs verbally.

### 8.2 Using the assessment and intervention document

- There is a separate section of the document for each intervention session, beginning at page 22. Intervention session 10 is the expected mid-point review, although this may vary according to the individual participant.
- At the top of the page, you should record:
  - The date of the home visit and the name and role of the clinician attending.
  - Consent from the participant or the consultee, as appropriate.
  - The time you set off from previous location for the visit, time you arrived at the visit, time you left, and time you returned to base. This will be used for future planning and costing.
- There is space for you to comment on the activities the participant has undertaken since the preceding visits. Check the project diary and discuss any incidents. Record any concerns or difficulties.
- Use the structured Activity Planning proforma to record activities undertaken at each visit and recommendations for activities to be performed by the participant between visits. See Section 8.3 for more details of how to complete this.
- Although the document is very structured, we have also provided additional space for you to make freehand clinical notes. You may also use your usual continuation sheets, but please make sure any additional sheets are attached and returned with the document.
- As this document must be returned to the study team at the end of the 12 weeks, you will also need to record your intervention as usual for your own team, using your own record system.

### 8.3 Activity planning

Activity planning will be used to create a clear plan for the participant to achieve their goals. The frequency of activities will be agreed with the MDT and with the participant. If the informal carer is willing and able and the team would like them to assist with activities, this should also be agreed in advance.

The example participant in table 5 has an overall goal of preparing all meals and drinks in the home. However, the participant needs to improve their standing balance, activity tolerance, and confidence to achieve this goal, as well as completing the activity directly relating to meals and drinks. Each activity clearly states the duration and frequency. There is a box to indicate which goal each activity relates to, to ensure they are strongly linked.

Activities will be reviewed at agreed intervals. For the below example, this will be reviewed in a week to see if the level needs to be progressed. Progression in relation to this goal may include adding in additional daily task of preparing a sandwich, increasing activity intensity, or modification of activity type. The duration and frequency of each activity may also be altered.

**Table 5: Example activity planning**

| **Name of activity to be undertaken** | **Linked goals** | **Follow up:**  **Has the patient undertaken the activity?**  **Y/N** | **Is the level to be progressed?**  **Y/N** |
| --- | --- | --- | --- |
| 1. To prepare 2 hot drinks a day for a week | 2 |  |  |
| 1. To complete daily exercises (sheets provided) after morning coffee – exercise sheets to be left next to kettle as reminder | 1 and 2 |  |  |
| 1. To fill kettle using small jug instead of taking to sink | 2 |  |  |
| 1. Remove loose rugs from kitchen | 2 |  |  |
| 1. To use perching stool when waiting for kettle to boil if required | 2 |  |  |

### 8.4 Making activities person-centred

When developing the action plan, recommended activities should be tailored to the individual participant’s goals. Use the assessment form to help develop these, paying attention to the section on likes and dislikes and the Compass of Life. If a specific area of need is identified, such as muscle weakness in specific muscle groups, rather than prescribing a generic ‘list of exercises’ or ‘practice tasks’, consider what type of activity to address the need this participant may be most interested in and therefore most likely to follow.

A participant is more likely to adhere to a tailored programme that is embedded into their everyday life, for example walking to the local shop for an item they regularly go for or walking the dog. The term ‘exercise’ may put some people off and they may be more easily engaged if they can relate to the activity; in essence, ensure that exercise has a ‘point’ to it. You might use improvements in length of time or record the number of repetitions for each activity to encourage and motivate participants by showing that they are progressing.

Some other examples of how you might incorporate preferences include planning dance elements into an intervention for a participant who enjoys music and dance, or linking walking practice and balance activities to the garden or greenhouse for a participant who enjoys gardening. Similarly, perching stools in the greenhouse may allow the participant to practice balance while sorting out pots or clearing up with opportunities to rest. Be inventive and creative so that what the participant finds enjoyable links into your plan, but always ensure they consent to your suggested plan.

#### 8.4.1 Keeping it functional

Activity plans may include a mix of both physical exercises and functional activities; some participants may prefer therapist supervised exercise programmes, while others may prefer to attend activity-based groups or to practice activities related to hobbies.

Functional activities could include encouragement to engage in community and social activities such as shopping and attending local activity groups. Informal carers will be encouraged to join in with activities where appropriate.

Exercise is included in nearly all effective therapeutic interventions for falls. Physical exercises could include strength and balance exercises and dual task exercises. The emphasis should be primarily on muscle strength and balance training to encourage healthy, active ageing rather than specifically on falls prevention. The aim should be for 150 minutes of moderate exercise per week for each individual but this will vary according to the participant’s general health and condition. Exercise should result in increased heart rate and breathing rate, but the participant exercising should be able to talk (not be gasping). For individuals with clinical conditions or specific considerations, accommodations should be made.

Positive health benefits of exercise are related to FITT-VP principles:

- **Frequency** (how often)
- **Intensity** (how hard)
- **Time** (duration/how long)
- **Type** (mode/what kind)
- **Volume** (total amount/quantity)
- **Progression** (advancement)

The following example shows how you might implement these principles with a participant wishing to walk further with the dog: start with once a day (**frequency**), on the flat (**intensity**), short sessions (**time**), for a set route, record the time taken and progress in one or more of the areas to show progression. When progressing, you could consider adding a hill into the walk or increase the distance (**intensity**) or perform the activity twice a day (**frequency**).

The therapist may need to supervise the functional task a few times during the intervention sessions to enable the activity to become embedded into the weekly plan. The informal carer may need support with introducing a functional activity into a daily routine and their needs should be considered throughout the intervention.

#### 8.4.2 Prompts and reinforcement

The recommendations for activity at each visit can be supported with printed information including pictures of any physical exercises to be carried out. **Please include copies of any printed advice or exercises you give to the participant when the folders are returned for analysis at the end of the study.** There are some links below to exercise leaflets that may be useful for people who have fallen.

However, providing a booklet and pictures in themselves may not be enough for a person with dementia. They are likely to need supported follow up, reinforcement and prompts, particularly when attempting to embed an activity into daily life. One potential prompt strategy is using cue cards placed in significant places around the participant’s home, using brightly coloured paper so they are easy to see, using words the participant uses, not jargon. However, like activities, prompts should also be tailored to the individual; informal carers may have good ideas for prompts and the best times for specific activity to be performed. Music may also be helpful used at set times of day to enable enjoyable exercises.

### 8.5 References

- The FITT-VP principles are available in an accessible format at this link: <http://www.csp.org.uk/sites/files/csp/secure/easy_exercise_guide_2016.pdf>
- The list of booklets available from Later Life Training includes postural stability strength and balance, Otago strength and balance (beginner, intermediate or advanced), chair based exercises, and backward chaining:

<https://www.laterlifetraining.co.uk/llt-home-exercise-booklets/> (These booklets may be printed or photocopied in their entirety without charge. If distributed as printed material, no charge must be made for this reproduction or provision without the permission of Later Life Training)

- The Chartered Society of Physiotherapy provides also useful information booklets; choose the one more suited to your participant:

<http://www.csp.org.uk/publications/were-talking-about-your-generation>

<http://www.csp.org.uk/publications/get-go-guide-staying-steady>

## 9 Six- and twelve-week reviews

### 9.1 Mid-point review

The mid-point review will be carried out by the key worker at week 6. This review is the opportunity to:

- Check if all the referrals from the MDT meeting have been acted on. If not, then follow up the referrals as appropriate and inform the member of the team who made the referral if there are any problems.
- Allow time to record the mid-point GAS scores on page 23 of the assessment document (see Section 7 of the manual for information on using the GAS). Participants may have completed their goals by this point. However, as the goals are an outcome measure for the study, they should not be changed or added to.
- Discuss the participant’s progress, offer praise for gains made as well as reassurance and motivation to continue. Thank them for taking part in the study so far.
- Review activity plans and discuss any progressions or new activities with the participant and, if appropriate, their informal carer. Ask them if there are any changes they would like to make.
- Review use of the activity diary and give prompts if needed to encourage the participant.
- Consider plans for progression and ongoing support, to allow for any referral waiting times.

At this stage, the participant may be able to self-manage for the next 6 weeks. If so, the key worker should set a date for the 12 week final assessment and encourage them to get in touch if they need any support or if things change. You may want to make a follow-up phone call in between the two reviews to see how they are. It is also possible that later sessions could focus more on support for the informal carer than on the participant’s activities. Some participants, however, will require ongoing visits throughout the 12 weeks.

The key worker should share the information gained from the mid-point review with the other therapists and support workers using the team’s usual methods of communication.

### 9.2 Twelve-week review

This can be completed by the physiotherapist or the occupational therapist depending on familiarity with the tests to be completed. At the 12-week review, you should:

- Review the period since the last intervention visit as usual. Comments on the past week should be added first to record any progress or relevant information on the recent progress or on any new incidents. Concerns or difficulties should be recorded as in previous sessions. Check through the activity diary.
- Complete the 12-week GAS scores on page 23 of the assessment document (see Section 7 of the manual for information on using the GAS)
- Complete the Timed Up And Go test. Record scores and comments for each part of the test as indicated on the form for week 12 (see page 16 of the manual for TUAG instructions)
- Complete a final discussion with the participant. This is really helpful information for planning further studies and to gain understanding of the participant’s experience of this type of intervention. Indicate if the informal carer has given a response or if it is the participant themselves; it is important to include the participant’s responses if possible. Record all comments on the following:
- What have they found useful or enjoyable?
- What could have been better?
- Which activities, if any, are they likely to continue? Note here if they have started to go to a regular groups or social activity since starting in the study
- Would they like to be referred to any ongoing services for progression (e.g. Staying Steady/community-based balance groups)? Make a note and refer on if this is required
- Any other comments they have about their experience
- Thank the participant and the informal carer involved in supporting them during the study
- Collect the participant’s files together ready for returning to the researchers for analysis. Remember to include copies of any printed advice guides or instructions given during the intervention period.
- Please leave the activity diary with the participant as they will need their diary to help them answer questions about what NHS treatments they have utilised over the last 12 weeks. The clinical researcher will collect the diary at their final outcome assessment visit.
- Following the final assessment, send the participant’s GP a summary of the interventions carried out and recommendations regarding ongoing service input where needed.

At the end of the twelve weeks, **thank you** to all therapy staff involved in delivering and devising the interventions!

Following the 12-week review with the therapist, the researchers will also follow up the participant to complete the quantitative and qualitative data collection. More information on this is available in Sections 11 and 12.

## 10 Consent, withdrawal and adverse events

### 10.1 Consent

Consent should be ongoing throughout the trial. At the start of each visit, check whether the participant understands that you will be doing some activities and is happy to continue with the session. If the participant does not seem to understand what is happening then you need to consider how consent was obtained for the trial. The initial documentation will state whether the participant gave consent themselves or whether a consultee gave consent for the participant. If a consultee has given consent then you may continue provided the participant seems to be happy to be doing the activities. If the participant gave initial consent themselves but no longer understands what is happening, we will need to seek a consultee to provide an opinion regarding the participant’s continuing participation in the trial. In this case, report the problem to the principal investigator at your site who will arrange this.

If, at any time, the participant seems unhappy or distressed by participating in the activities then do not continue, regardless of whether consent has been obtained. You may ask whether the participant would be willing to continue another day. If the participant does not wish to continue another day then ask them if they would like to withdraw from the trial.

### 10.2 Withdrawal

Participants are free to withdraw at any time. You may ask them if they would like to give a reason but they do not have to do so. They may choose to withdraw from the intervention but still complete the final assessment and/or a qualitative interview. Please discuss the various options with them so that you can inform the PI of their preference.

If the participant would like to withdraw, please record this in the clinical notes along with any reason, if given. Please inform the principal investigator and return the trial notes to the principal investigator.

### 10.3 Adverse events

An adverse event is any untoward medical occurrence in a study participant, not necessarily caused by the intervention. This might include falling, fainting, or intercurrent illness. It is important that we know about adverse events during the trial period, but we need to balance expected and insignificant events versus those that are significant. Some adverse events may be expected given the frailty of the participant population but should still be reported. Table 6 below gives examples.

**Table 6: Examples of adverse events**

| **Do not report** | Tiredness | Expected, insignificant |
| --- | --- | --- |
|  | Minor muscle fatigue |  |
| **Do report** | Chest infections; urinary tract infections; delirium | Adverse Event |
|  | Injurious falls |  |
|  | Any event that results in contact with healthcare professionals |  |
|  | Hospital admission | Serious Adverse Event |
|  | Permanent disability or incapacity |  |
|  | Life-threatening events |  |
|  | Death |  |

Please report any adverse events to your site principal investigator who will complete the relevant paperwork. If you are not sure whether an adverse event is significant enough to be reported then discuss it with your principal investigator who will make a decision. Serious Adverse Events need to be reported as soon as possible.

If an adverse event requiring immediate medical attention occurs during your visit, please follow your usual protocols for obtaining medical attention and inform the principal investigator afterwards.

## 11 Final participant assessment by research team

At 12 weeks, the clinical researcher will carry out a second visit to repeat most of the outcome measures completed at the baseline assessment with the participant and informal carer (see Table 1 on page 10). The exception is the MOCA; completing this at baseline will enable us to describe the cognitive function of participants, but it will not be repeated as the intervention is not expected to have an impact on cognition.

One additional measure – the Health Utilisation Questionnaire (HUQ) – will be completed at the final participant assessment. This will collect information on the health and social care services used by the participant during the intervention period. The participant will use their diary to help them answer this questionnaire. The diary will be collected by the clinical researcher at this visit.

We will include a small number of open questions at the final follow-up interview to explore participants’ views on the measures used and to identify any additional outcomes of the intervention that have not been captured. In addition, some participants and informal carers will be invited to take part in the process evaluation (see next section).

## 12 Process evaluation

The pilot study will include what is known as a ‘process evaluation’. This is a way of assessing how well an intervention is working and if it is being implemented in the most effective way. Data will be collected throughout the intervention to understand why and how it works (or does not work) in practice. The process evaluation will focus on participants’, informal carers’ and professionals’ experiences and views on the DIFRID intervention, and your participation will be crucial to its success.

### 12.1 Your role

As the clinicians delivering the intervention, we will need your help to identify any feasibility issues or adaptations needed before proceeding to a full trial. We will collect data through documentary analysis, interviews, informal discussions, observation, and audio recordings of intervention delivery and meetings.

#### Documentary analysis

Some information will be collected by reviewing the assessment and intervention documents. You are also asked to send a copy of all referral letters to the qualitative team so that we can look at the impact of the intervention on referral patterns. Please include a brief paragraph in your referral letters to explain that the professional you refer to may be contacted and invited to take part in an interview.

#### Observation and recording of intervention delivery

Other information will be collected through observation of intervention sessions and audio recordings of intervention delivery and meetings. We will be as flexible as possible in negotiating opportunities for observation, as we do not aim to be burdensome to staff delivering the intervention or other participants, and we do not wish to influence the delivery of the intervention. We will aim to be unobtrusive and would withdraw if our presence was having a negative effect on the interaction. Observation of assessment and intervention sessions will only take place with the permission of the participant and informal carer.

#### Interviews and focus groups

You may also be invited to take part in informal discussions, interviews and/or focus groups with the qualitative researchers. We hope that as many staff as possible will agree to participate. This will ensure that those most closely involved in delivering the intervention have opportunities to share their experiences and opinions regarding the DIFRID intervention and to provide feedback on what works and what needs to be changed. Interviews with clinicians delivering the intervention will include discussion of the intervention itself and associated materials (e.g. this manual, initial training, and assessment documents). Interviews with MDT members will explore the perceived value and sustainability of the MDT meetings as part of the intervention. Alternative models of obtaining specialist input will also be explored. Interviews with other associated professionals, such as those who work at services to which participants were referred, will also be carried out.

All interviews and focus groups will be audio-recorded and transcribed for analysis. The transcripts will be anonymised to remove identifying details such as names and locations. Although quotations would be used in disseminating the findings, these will be attributed only by study ID number and role (e.g. physiotherapist).

### 12.2 The role of participants and informal carers

Interviews will be arranged with participants and informal carers to explore the acceptability and perceived value of the intervention. We will also explore the extent to which participants felt the intervention was tailored, their views on the intensity of the intervention and staff involved in delivering the intervention, and any suggested changes to the intervention. We will also review the project diaries completed by participants and informal carers

### 12.3 Consent to participate in the process evaluation

While informed consent will be sought from PWD and informal carers regarding their participation in the process evaluation, we will not seek written consent from staff delivering the intervention since their participation is an integral part of their role. We do not require that staff must agree to all requests for observation or interview by the research team. However, there is an expectation that staff agreeing to deliver the intervention will be willing to contribute to the process evaluation.

## Appendix 1: Contact details of research team

### Newcastle University

| Dr Louise Allan | Chief Investigator | 0191 208 1336 | [Louise.allan@ncl.ac.uk](mailto:Louise.allan@ncl.ac.uk) |
| --- | --- | --- | --- |
| Beth Edgar | DIFRID Secretary | 0191 208 1314 | [beth.edgar@ncl.ac.uk](mailto:beth.edgar@ncl.ac.uk) |
| Claire Bamford | Qualitative Lead | 0191 208 7047 | [claire.bamford@newcastle.ac.uk](mailto:claire.bamford@newcastle.ac.uk) |
| Alison Wheatley | Qualitative Researcher | 0191 208 5147 | [alison.wheatley@ncl.ac.uk](mailto:alison.wheatley@ncl.ac.uk) |
| Liz Flynn | DIFRID Physiotherapist |  | [Elizabeth.Flynn@nuth.nhs.uk](mailto:Elizabeth.Flynn@nuth.nhs.uk) |
| Amy Smith | DIFRID Occupational Therapist | 07900 606295 | [amy.smith16@nhs.net](mailto:amy.smith16@nhs.net) |

### Newcastle upon Tyne Hospitals NHS Foundation Trust

| Vicki Hetherington | Senior Clinical Trials Officer | 0191 208 1351 | [Victoria.Hetherington@ntw.nhs.uk](mailto:Victoria.Hetherington@ntw.nhs.uk) |
| --- | --- | --- | --- |

### North Tees and Hartlepool NHS Foundation Trust

| Laura O’Rourke | Research Nurse | 01642 383204 | [Laura.ORourke@nth.nhs.uk](mailto:Laura.ORourke@nth.nhs.uk) |
| --- | --- | --- | --- |

### Norfolk Community Health and Care NHS Trust

| Steph Tuck | Research OT and Falls Lead | 01603 272251 | [Stephanie.Tuck@nchc.nhs.uk](mailto:Stephanie.Tuck@nchc.nhs.uk) |
| --- | --- | --- | --- |

## Appendix 2: Case report form

## Appendix 3: Assessment document Intervention sessions 3 to 9 (pages 27-40 of the assessment document) are the same as Intervention session 2 above.

##

## Intervention sessions 12 to 21 (pages 45-64 of the assessment document) are the same as Intervention session 11 above.

## Appendix 4: Consensus Statements

| **Statement** | **Outcome** | **Round** | **Percentage** | **Final Selection** |
| --- | --- | --- | --- | --- |
| Feasibility, design and inclusion criteria of the study |  |  |  |  |
| The brief requires us to design a complex intervention. | Agreed | 1 | 92.9 | As statement |
| Patients with non-injurious falls should be eligible for the intervention | Agreed | 1 | 100.0 | As statement |
| Fallers with an acute medical illness causing their fall, e.g. pneumonia or stroke, are included | No consensus | 2 | 46.2 | Include |
| Fallers should be recruited either within 1 week of the fall or 1 month of the fall | No consensus | 2 | 53.8, 46.2 | 1 month |
| A feasible and useful sample size would be (% given for range of choices up to 30 participants) | No consensus | 1 | 64.3 | up to 30 participants |
| The number of sites included should be 3 sites. | Agreed | 2 | 76.9 | 3 sites |
| It is feasible to recruit to WP4 | Agreed | 2 | 100.0 | As statement |
| Setting of the study |  |  |  |  |
| It would be useful to recruit participants presenting with a fall in the ED | Agreed | 1 | 92.9 | As statement |
| It would be useful to recruit participants presenting with a fall to paramedics if single ambulance stations are targeted | Agreed | 1 | 92.9 | As statement |
| It would be useful to recruit participants presenting with a fall in the primary care setting | Agreed | 1 | 85.8 | As statement |
| If we are recruiting participants who have had a fall within the last week, it would be useful for GPs to write to all patients on their QOF dementia register | No consensus | 2 | 30.8 | Rejected as we will not be recruiting patients up to one week after a fall |
| If we are recruiting participants who have had a fall within the last month, it would be useful for GPs to write to all patients on their QOF dementia register | Agreed | 2 | 84.6 | As statement |
| 14. It would be useful to recruit participants in another setting. | Agreed | 1 | 78.6 | As statement |
| Mean priorities for alternative settings |  |  |  |  |
| Community services e.g. multidisciplinary outreach teams |  |  | 3.1 | Include |
| Domiciliary physiotherapy |  |  | 4.6 | Exclude |
| Supported discharge teams |  |  | 3.4 | Include |
| Telecare services |  |  | 3.4 | Include |
| Social services re-enablement teams |  |  | 4.4 | Exclude |
| Memory clinics |  |  | 4.4 | Exclude |
| Dementia cafes |  |  | 5.5 | Exclude |
| Social media |  |  | 7.2 | Exclude |
|  |  |  |  |  |
| The intervention should primarily take place in the patient's home | Agreed | 1 | 85.7 | As statement |
| The setting of the intervention should make use of existing pathways only when referral from the team deems it would be useful for the individual | Agreed | 1 | 85.7 | As statement |
| Content of the intervention (staff) |  |  |  |  |
| A Physiotherapist should be routinely involved | Agreed | 1 | 71.4 | As statement |
| An Occupational therapist should be routinely involved | Agreed | 1 | 71.4 | As statement |
| A Geriatrician should be routinely involved via multidisciplinary team meeting and available for face to face consultation if required | No consensus | 2 | 61.5 | As statement |
| A Rehabilitation support worker should be routinely involved | Agreed | 1 | 71.4 | As statement |
| A Registered general nurse should be routinely involved via multidisciplinary team meeting and available for face to face consultation if required | No consensus | 2 | 61.5 | As statement |
| A Community psychiatric nurse should be available on referral | Agreed | 1 | 71.4 | As statement |
| A Social worker should be available on referral | Agreed | 1 | 71.4 | As statement |
| Re-enablement workers should be available on referral | Agreed | 1 | 71.4 | As statement |
| An Old Age Psychiatrist should be available on referral | Agreed | 2 | 84.6 | As statement |
| A Podiatrist should be available on referral | Agreed | 2 | 92.3 | As statement |
| Content of the intervention (assessment) |  |  |  |  |
| Assessment should involve multiple sources of information including information from carers | Agreed | 1 | 100.0 | As statement |
| Assessment should include direct observation | Agreed | 1 | 100.0 | As statement |
| Formal assessments of gait and balance should be carried out by the Timed Up and Go test | No consensus | 2 | 61.5 | As statement |
| A home hazard assessment should include a walk around the house to determine where actual falls have occurred and negotiate how these might be reduced | Agreed | 1 | 92.9 | As statement |
| An assessment of comorbidities is required | Agreed | 1 | 100.0 | As statement |
| An osteoporosis risk assessment is required | Agreed | 1 | 92.9 | As statement |
| A vision assessment is required | Agreed | 1 | 100.0 | As statement |
| A medication review is required | Agreed | 1 | 100.0 | As statement |
| All patients require attendance for a lying and standing BP | No consensus | 2 | 53.8 | As statement : to be carried out by therapist in the patient's home |
| A continence assessment is required | Agreed | 1 | 78.6 | As statement |
| An assessment of challenging behaviour is required | Agreed | 1 | 92.9 | As statement |
| Tools which assess non-verbal signs of pain should be used | Agreed | 1 | 92.9 | As statement |
| A multidisciplinary team meeting should be available if needed | Agreed | 1 | 92.9 | As statement |
| Carer stress should be routinely assessed | Agreed | 1 | 92.9 | As statement |
| Content of the intervention (methodology and quantity) |  |  |  |  |
| Interventions should be based on goals set by the patient and carer | Agreed | 1 | 85.7 | As statement |
| Therapists should work with service users to minimise the risk of falling, as this may improve confidence and enable realistic risk taking. | Agreed | 1 | 100.0 | As statement |
| Therapists should facilitate caregivers, family and friends to adopt a positive approach to risk | Agreed | 1 | 100.0 | As statement |
| Exercise interventions should be informed by evidence based formats such as the Otago programme but tailored to the circumstances of PWD and embedded in their daily life | Agreed | 2 | 69.2 | As statement |
| The total number of physiotherapy sessions available in the first 3 months (including sessions delivered by a support worker) should be 16, 20 or 24 | No consensus | 2 | 30.8, 38.5, 30.8 | 20 sessions: Twice weekly (weeks 0-8) tapering to once weekly (weeks 9-12) |
| The total number of occupational therapy sessions available in the first 3 months should be 3-4 | No consensus | 2 | 61.5 | 4 |
| Therapists should offer service users information on assistive devices and facilitate delivery | Agreed | 1 | 100.0 | As statement |
| Therapists should help the service user and caregiver to develop a meaningful programme of activities | Agreed | 1 | 100.0 | As statement |
| Therapists should undertake observed activities with the service user to facilitate new learning | Agreed | 1 | 92.9 | As statement |
| Intervention staff should be able to provide basic carer education & support, referring to other agencies as needed | Agreed | 2 | 76.9 | As statement |
| Staff training |  |  |  |  |
| Tier 2 training is required for intervention staff | Agreed | 2 | 84.6 | As statement |
| Training needs to include how to tailor an intervention for PWD | Agreed | 1 | 100.0 | As statement |
| Training needs to include advice on how to engage and motivate PWD | Agreed | 1 | 100.0 | As statement |
| Training should include on the job role modelling | Agreed | 1 | 100.0 | As statement |
| Outcome measures for the intervention |  |  |  |  |
| The primary outcome measure be a numerical measure of falls | Agreed | 2 | 76.9 | As statement |
| Secondary outcomes should include health related quality of life measure | Agreed | 1 | 100.0 | As statement |
| The best health related quality of life measure would be Quality of life in Alzheimer’s disease (QOL-AD) | Agreed | 2 | 69.2 | As statement |
| Secondary outcomes should include activities of daily living measure | Agreed | 1 | 92.9 | As statement |
| The best activities of daily living measure would be Disability Assessment for Dementia (DAD) | Agreed | 2 | 84.6 | As statement |
| Secondary outcomes should include carer burden measure | Agreed | 1 | 92.9 | As statement |
| The best carer burden measure would be Zarit Burden interview | Agreed | 2 | 69.2 | As statement |
| Secondary outcomes should include psychological consequences of falling measure | Agreed | 1 | 85.7 | As statement |
| The best psychological consequence measure e.g. fear of falling would be the Modified Falls Efficacy scale | Agreed | 1 | 71.4 | As statement |
| Secondary outcomes should include physical activity measure | No consensus | 1 | 64.2 | As statement |
| The best physical activity measure would be a wearable physical activity monitor | Agreed | 1 | 78.6 | As statement |
| Secondary outcomes should include Strength and balance measure | No consensus | 1 | 57.1 | As statement- this would be TUG as in initial assessment |
| Secondary outcomes should include goal setting or performance measure | No consensus | 1 | 35.7 | As statement |
| The best goal setting or performance measure would be Goal Attainment scaling | Agreed | 2 | 84.6 | As statement |
| The best carer quality of life measure would be EQ-5D- 5L | No consensus | 1 | 57.1 | Exclude- see below |
| The most popular Carer quality of life measure was EQ-5D-5L, but it was suggested that a measure of carer burden would be sufficient. | No consensus | 2 | 53.8 | As statement |
|  |  |  |  |  |
| Prioritise the remaining domains where consensus was not achieved (1 highest - 4 lowest) |  |  |  |  |
| Goal setting measure |  | 2 | 2.0 | include |
| Physical activity measure |  | 2 | 2.5 | include |
| Strength and balance measure |  | 2 | 2.5 | include |
| Carer quality of life |  | 2 | 3.0 | exclude |
|  |  |  |  |  |

This research is funded by the National Institute for Health Research Health Technology Assessment Programme (Project number 13/78/02). Further information is available at: <http://research.ncl.ac.uk/difrid/> . This document presents independent research commissioned by the National Institute for Health Research (NIHR). The views and opinions expressed by authors in this publication are those of the authors and do not necessarily reflect those of the NHS, the NIHR, MRC, CCF, NETSCC, the Health Technology Assessment Programme or the Department of Health.
