# Supplementary material for: Is it feasible to deliver a complex intervention to improve the outcome of falls in people with dementia? A protocol for the DIFRID feasibility study
Source: Pilot Feasibility Stud. 2018 Nov 10;4:170. doi: 10.1186/s40814-018-0364-7 (PMC6230281; doi:10.1186/s40814-018-0364-7)
Supplement: Supplementary file 5 — Topic guides for the Process Evaluation. (DOCX 29 kb) [file 40814_2018_364_MOESM5_ESM.docx]

**Topic guides for process evaluation study**

**Staff responsible for recruitment and assessment of outcomes**

Introduction (Introduce self & explain aspects of interview process)

- Please don’t feel the need to be polite or restrained; we need your honest feedback and suggestions. Reassure re confidentiality.

Can you start by telling me about how the recruitment process has worked in practice?

- What has been successful?
- What have the main challenges been?

How interested do patients seem to be in the intervention?

- What strategies have you used when explaining the study to patients and trying to engage them?
- From your perspective, what are the facilitators and barriers to getting people/patients engaged in the study?
- What else could we do to make the intervention more appealing to patients and carers?

Have there been any patients who met the inclusion criteria but you felt were not appropriate for the study?

- Would you suggest any changes to the inclusion/exclusion criteria?

What sense do you have of how feasible it would be to proceed to a full trial with a control group etc?

- Are there any changes you would recommend to recruitment processes?
- Was there any additional support you needed to recruit patients?
- Was there anything in the recruitment materials (PIS, consent forms) that could be improved?

Can you tell me about how the assessment processes and outcome measures have worked in practice?

- From your contact with patients and carers, to what extent do you feel the outcome measures are capturing the difference that the intervention has made to their lives?

What sense do you have of how useful the intervention is?

When we were designing the intervention, we developed some theories about how we thought it would work. (describe theories from realist review) Does this line up with your experience?

**Staff responsible for developing the intervention and training and supervising intervention delivery**

How do you think the training went?

- What worked well?
- What might you do differently in the future?
- In terms of taking this work forward, how could we improve the initial training session(s)?

How has the supervision process been?

- How frequently have you met?
- What have been the main issues raised in supervision?
- Is there anything that could be improved?

How do you think the study/intervention is going so far?

How confident are you in the intervention?

What reservations do you have about the intervention?

Which aspects of the DIFRID intervention do you feel most confident with/have been most useful*?

Which aspects of the DIFRID intervention do you find most challenging/have been least useful*?

Overall, what sense do you have of how useful the intervention is?

In this study, the DIFRID intervention is delivered by physiotherapists, occupational therapists and rehabilitation assistants. From your perspective what are the advantages and disadvantages of using staff with this skill mix?

What kinds of patients do you think would benefit most from this type of intervention? Are there patients for whom it would not be useful?

Based on feedback from supervision, how interested do patients seem to be in the intervention?

From your perspective, what are the facilitators and barriers to getting people/patients engaged in the study?

From your perspective, what are the facilitators and barriers to implementing the intervention?

From your perspective, what are the facilitators and barriers to evaluating the acceptability and impacts of the DIFRID intervention?

Are there any changes we should make to the DIFRID intervention?

Is there anything else that we haven’t covered about the DIFRID intervention?

When we were designing the intervention, we developed some theories about how we thought it would work. (describe theories from realist review) Does this line up with your experience?

**Staff delivering the intervention**

What are/were your expectations about the DIFRID intervention?

How do you think the study/intervention is going so far?

How confident are you in the intervention?

What reservations do you have about the intervention?

Which aspects of the DIFRID intervention do you feel most confident with/have been most useful*?

Which aspects of the DIFRID intervention do you find most challenging/have been least useful*?

What opportunities have you had to discuss the value of the intervention with your colleagues?

How might we modify the DIFRID intervention?

From your perspective, what are the facilitators and barriers to implementing the intervention?

Overall, what sense do you have of how useful the intervention is?

In this study, the DIFRID intervention is delivered by physiotherapists, occupational therapists and rehabilitation assistants. From your perspective what are the advantages and disadvantages of using staff with this skill mix?

What kinds of patients do you think would benefit most from this type of intervention? Are there patients for whom it would not be useful?

Based on feedback from supervision, how interested do patients seem to be in the intervention?

From your perspective, what are the facilitators and barriers to getting people/patients engaged in the study?

Could you describe the process of tailoring the intervention to the individual patient?

- How well did you think this worked in practice?
- What were the facilitators and barriers to tailoring and embedding?

How helpful were different components of the intervention (e.g. training, manual, MDT meetings, supervision)?

Are there any changes we should make to the intervention materials (e.g. the assessment form)?

From your perspective, how well did the intervention ‘fit’ with other services?

Do you feel you have the support you need to deliver the intervention?

What dementia training had you previously received?

How do you think the training went?

- What worked well?
- What do you think could have been done differently?

How well has the supervision process gone?

- Is there anything that could be improved?
- Was there any training or support that you felt you needed but didn’t receive?

In terms of taking this work forward, how could we improve the initial training session(s)?

How will your experience with the DIFRID intervention influence your usual practice in the future?

Is there anything else that we haven’t covered about the DIFRID intervention?

When we were designing the intervention, we developed some theories about how we thought it would work. (describe theories from realist review) Does this line up with your experience?

**Members of the multidisciplinary team**

What are/were your expectations about the DIFRID intervention?

How do you think the intervention is going so far?

How confident are you in the intervention?

What reservations do you have about the intervention?

Which aspects of the DIFRID intervention do you feel most confident with/have been most useful*?

Which aspects of the DIFRID intervention do you find most challenging/have been least useful*?

From your perspective, what are the facilitators and barriers to implementing the intervention?

Overall, what sense do you have of how useful the intervention is?

If we were taking the intervention forward, how might you change it?

In this study, the DIFRID intervention is delivered by physiotherapists, occupational therapists and rehabilitation assistants. From your perspective what are the advantages and disadvantages of using staff with this skill mix?

What kinds of patients do you think would benefit most from this type of intervention? Are there patients for whom it would not be useful?

From your perspective, what are the facilitators and barriers to getting people/patients engaged in the study?

What have been the benefits of including an MDT in the intervention?

- For patients/carers?
- For staff delivering the intervention?
- For yourself?

How well did the MDT meetings work in practice/logistically?

Are there any changes we should make to the MDT process?

Is there anything else that we haven’t covered about the DIFRID intervention?

**Health and social care professionals involved in the care of patients receiving the intervention**

(Professionals to whom referrals have been made as part of the DIFRID intervention, or who were delivering services to the patient or carer during the intervention period)

We are interested in how the DIFRID intervention fits with other existing services.

Can you tell me about your involvement with <*name>*?

Did you have any contact with staff delivering the DIFRID intervention?

- Can you tell me a bit more about that?

Did the fact that <*name>* was also receiving the DIFRID intervention impact on your work at all?

- Time constraints
- Overlap/conflicting advice

In this study, the DIFRID intervention is delivered by physiotherapists, occupational therapists and rehabilitation assistants. From your perspective what are the advantages and disadvantages of using staff with this skill mix?

From your perspective, what are the facilitators and barriers to implementing the intervention?

Overall, what sense do you have of how useful the intervention is?

How might we modify the DIFRID intervention?

Is there anything else that we haven’t covered about the DIFRID intervention?

When we were designing the intervention, we developed some theories about how we thought it would work. (describe theories from realist review) Does this line up with your experience?

**Patients receiving the intervention**

Did you feel this was a good intervention for you?

- Tell me more about that

What did you like about the intervention sessions?

What did you dislike? What could have been different?

Which aspects of the DIFRID intervention have been most useful?

Which aspects of the DIFRID intervention have been least useful?

How did you feel about the activities that you were asked to do? (Were they personalised enough?)

Has the intervention made any difference to you?

- Mobility
- Confidence
- Activities
- Number of falls
- Anything else?
- Are there any areas you were hoping the intervention would improve that haven’t improved?

Thinking about the intervention materials such as the diary and the manual, are there any changes that you think we should make?

Could you tell me a bit about the staff delivering the intervention?

- Were they knowledgeable?
- How well did you think they communicated and interacted with you?

Can you tell me a bit about the goal you have been working towards?

- How did you chose that as a goal?
- How much progress have you made?
- How did you feel about the process of setting goals?
- Do you feel you have as much help as you need to help you achieve your goals?
- What else would help?

What kinds of patients do you think would benefit most from this type of intervention? Are there patients for whom it would not be useful?

Is there anything else that we haven’t covered about the DIFRID intervention?

**Family members of patients receiving the intervention**

What were/are your expectations about the DIFRID intervention?

Did you feel this was a good intervention for <*name*>?

- Tell me more about that
- What did you like about the intervention sessions?
- What did you dislike? What could have been different?
- How engaged did <*name>* seem to be in the intervention?
- Which aspects of the DIFRID intervention have been most useful?
- Which aspects of the DIFRID intervention have been least useful?
- How did you feel about the goals that *<name>* has been working towards?

Has the intervention made any difference to <*name>*?

- Mobility
- Confidence
- Activities
- Number of falls
- Anything else?
- Are there any areas you were hoping the intervention would improve that haven’t improved?

What about yourself, how involved have you been in the intervention?

- Were you satisfied with that level of involvement?
- Did you receive any education or training? Was it useful?
- Has the intervention had any impacts on you?
- Is there anything we could have done differently to help you?

Thinking about the intervention materials such as the diary and the manual, are there any changes that you think we should make?

The DIFRID intervention is delivered by physiotherapists, occupational therapists and rehabilitation assistants. From your perspective what are the advantages and disadvantages of using staff with this skill mix?

Could you talk about your perception of the staff delivering the intervention?

- Were they knowledgeable?
- How well did you think they communicated and interacted with you?

What kinds of patients do you think would benefit most from this type of intervention? Are there patients for whom it would not be useful?

Is there anything else that we haven’t covered about the DIFRID intervention?

When we were designing the intervention, we developed some theories about how we thought it would work. (describe theories from realist review) Does this line up with your experience?
